# The effect of 5-hydroxytryptophan, a serotonin precursor, on adults with high levels of Attention Deficit Hyperactivity Disorder traits: A randomised, controlled trial

**DOI:** 10.1371/journal.pone.0349512

**Published:** 2026-05-20

**Authors:** Eleanor Jackson, Timothy Riley, Paul G. Overton

**Affiliations:** School of Psychology, University of Sheffield, Western Bank, Sheffield, United Kingdom; Hochschule Niederrhein - Campus Mönchengladbach: Hochschule Niederrhein - Campus Monchengladbach, GERMANY

## Abstract

**Background:**

Although several effective therapies exist for Attention Deficit Hyperactivity Disorder (ADHD), current pharmaceutical treatment carries a high risk of misuse and high levels of discontinuation, evidencing a need for alternatives. One possible avenue is serotonergic intervention, particularly within the serotonin synthesis pathway, where there are several potential loci of dysfunction in ADHD. This study aimed to assess the efficacy of acute 5-hydroxytryptophan, a serotonin precursor, in reducing distractibility in adults with high levels of ADHD traits.

**Trial design and methods:**

The study consisted of a randomised, controlled trial to assess the effects of acute 5-hydroxytryptophan administration compared to placebo. Participants consisted of individuals with high (N = 56) and low (N = 56) levels of ADHD traits, determined using the Adult ADHD self-report scale screener (ASRS v1.1), with randomised allocation to intervention or placebo in a 1:1 ratio. Both participants and investigators delivering the trial were blind to the allocation. Baseline testing of distractibility using a task-relevant (Eriksen flanker) and novel task-irrelevant (N-back coupled with an auditory stimulus) paradigm was completed, participants were given the intervention or placebo, and tasks were repeated 90 min post-administration.

**Results and conclusions:**

The flanker and N-back task found few differences between individuals with high and low levels of ADHD traits, and the N-back task did not produce a distractor effect as initially predicted. 5-hydroxytryptophan produced no significant positive effect in any measure of distractibility that differed between individuals with high or low ADHD traits. Only accuracy on silent trials in the N-back task was affected by 5-hydroxytryptophan administration: placebo participants showed an improvement in performance and 5-hydroxytryptophan-administered participants did not. 19.6% of participants in the 5-hydroxytryptophan group experienced adverse events of fatigue, nausea or vomiting. Although 5-hydroxytryptophan did not elicit a positive effect on ADHD traits,further work should be conducted with measures more sensitive to ADHD traits to fully understand the impact of 5-hydroxytryptophan supplementation on the condition.

## Introduction

Attention Deficit Hyperactivity Disorder (ADHD) is a common and pervasive disorder affecting around 5% of children and 2.5% of adults worldwide [1–3]. The disorder presents with key traits of inattention, hyperactivity and impulsivity, alongside functional impairments that result in a lower quality of life and, ultimately, a lower life expectancy [4,5]. Although ADHD is widely treated, current gold-standard psychostimulants produce only a 30% or 50% reduction in ADHD traits, and an estimated 10–40% of people are non-responders [6–8]. Psychostimulant use also poses a significant elevation in risk for cardiovascular diseases, with each year of cumulative use being associated with a 4% increase in risk [9]. Further controversy surrounds the abuse of psychostimulants; the 2018 USA National Survey on Drug Use and Health reported 5.1 million people over the age of 12 had misused psychostimulant products [10]. Stimulants for ADHD are frequently diverted or shared with others, with one survey of a college population revealing 61.7% of respondents prescribed ADHD medicines had diverted medication at least once, and a further study found 54% of stimulant users were approached by others to divert their medication [11,12]. In the UK, there are currently two non-stimulant drugs available for the treatment of ADHD, atomoxetine and guanfacine, but both are offered only as a secondary line of treatment and have been found to be less effective in managing ADHD traits than their psychostimulant counterparts [13,14].

Given the complex and controversial nature of psychostimulant efficacy and use, there is a significant desire for complementary and alternative therapies; one recent survey found 76% of adults with ADHD sampled had initiated use of a complementary or alternative medicine outside of the use of a medical professional’s recommendation [15,16]. Such desires need to be reflected in research to provide evidence-based therapeutic options for people with ADHD.

One area of pharmacological interest is the serotonergic system, a member of the monoaminergic family of neurotransmitters, which is robustly observed as dysfunctional in ADHD [17–20]. More recently, consideration of metabolic dysfunction has gained traction; serum levels of serotonin have been observed to be reduced in individuals with ADHD across several studies [21–23]. Furthermore, decreased quantities of metabolites downstream of serotonin, and increased amounts of metabolites in the major metabolic pathway of tryptophan, the amino acid from which serotonin is formed, suggest an underperforming serotonergic system in ADHD [24–26]. Some of these changes to metabolite levels have also been correlated with performance in continuous performance tasks [27,28].

Much of the work looking at serotonin synthesis within ADHD focuses on the initial intake of tryptophan rather than the later formation of serotonin, likely due to the ease with which tryptophan intake can be studied. A recent systematic review highlighted that most studies looking at tryptophan levels in ADHD found ADHD cohorts to have elevated rather than depleted levels of tryptophan [26]. Despite the evidence that the serotonin system is underperforming in ADHD, some studies have used the elevated levels of tryptophan as a launchpad to look at the effects of tryptophan loading. Tryptophan depletion has mixed effects on attention [29]. Likewise, a randomised, controlled trial assessing tryptophan loading and its impact on attention and impulsivity found no effect on any measure of ADHD traits in both medicated and unmedicated individuals with ADHD [30]. Such evidence suggests that a problem with tryptophan uptake may be one driver of reduced serotonin levels in ADHD.

Another possibility is a problem with the conversion of tryptophan to 5-hydroxytryptophan (5-HTP), the precursor to serotonin. Tryptophan hydroxylase 2 (TPH2), the enzyme catalysing this conversion and the rate-limiting step in serotonin synthesis, has several variants which have been observed as preferentially transmitted in ADHD probands [31–34]. Such variants may reduce the production of 5-HTP, resulting in reduced serotonin production [35,36]. Further evidence from neurometabolic disorders such as phenylketonuria, a condition which presents with ADHD traits, has found improvements in cognitive function from 5-HTP supplementation [37–39]. As such, it seems pertinent to investigate the possible therapeutic benefits of 5-HTP supplementation, avoiding potential problems with the uptake of tryptophan and TPH2 [20].

Serotonins’ involvement in ADHD may link to its role in the regulation of attention and distractibility, particularly in key loci such as the superior colliculus, a structure responsible for eye and head movement in response to sensory stimuli, especially novel or unexpected stimuli [40–42]. Evidence suggests that the superior colliculus may be hyper-responsive to sensory stimuli in ADHD [43,44]. Pre-clinical work has suggested a possible link between serotonin transmission and collicular sensory hyper-responsivity. Significant serotonergic projections terminate in the superior colliculus, and serotonin application to the tissue suppresses visual responses [45,46], suggesting that sensory hyper-responsiveness may result from reduced collicular serotonin. In turn, psychostimulant drugs may derive part of their therapeutic efficacy by raising collicular serotonin levels, since in vitro application of d-amphetamine and methylphenidate depresses stimulus intensity in the superior colliculus, which can be blocked with the application of serotonin antagonist metergoline [47].

As serotonin plays a core role in distractibility, it seems pertinent to focus on changes in distractibility in the development of serotonin-based therapeutics. Distractibility is considered by many to be one of the most common traits of ADHD, and along with inattention is the most likely ADHD trait to persist into adulthood, suggesting it is a significant issue to be tackled [48,49]. It is well accepted that ADHD is highly heterogeneous, with diagnoses falling into three subcategories based on symptom experiences [50]. However, current assessment of ADHD therapies using reduction in traits as assessed by scores on behavioural questionnaires [7] may miss some of the nuances or symptom-specific improvements that certain interventions yield. As such, a more helpful approach to therapy development may be to focus on specific symptom domains, such as distractibility, which would result in more personalised, specific therapies based on need [44].

Considering the existing research on serotonin synthesis, ADHD, attention and current pharmacotherapies, it is possible that a reduction in 5-HTP synthesis may be a cause of reduced serotonin in ADHD. Mechanistically, this may reduce serotonergic inhibition in the superior colliculus, resulting in increased response to stimuli and increased distractibility. Furthermore, we postulate that 5-HTP supplementation may ameliorate traits of ADHD as a result and could thus be a promising nutraceutical intervention for the condition. Limited research has been conducted on the impact of 5-HTP on attention and distractibility, with only one study on rhesus macaques completed. This research, however, demonstrated that 5-HTP modulated attention in these animals in a bidirectional manner, dependent on the macaque’s baseline levels of attention; those with low initial attention had an increase in looking behaviour, those with high initial attention experienced a decrease [51]. At the present time, no trials have looked at the efficacy of 5-HTP in the treatment of ADHD, but several systematic reviews of 5-HTP treatment for other conditions do exist. A review of trials data for 5-HTP treatment of depression found poor quality evidence, but the available evidence did suggest better performance than placebo at treating depression [52]. The review reported some side effects and a potential link to the development of Eosinophilia-Myalgia syndrome; however, a more recent review found only mild adverse effects linked to 5-HTP administration in fibromyalgia treatment, and a host of other trials have found 5-HTP to be safe and tolerable [52–55].Furthermore, no cases of 5-HTP use have been linked to Eosinophilia-myalgia in recent years [53,54]; 5-HTP is considered safe for use and is widely available as a nutraceutical product, making it an accessible option for treatment if beneficial for ADHD traits.

As such, this research intends to explore the impact of acute 5-HTP administration on distractibility across human populations with high and low levels of ADHD traits. We hypothesise that performance on task-relevant and task-irrelevant distractor tasks will be lower for participants with higher levels of ADHD traits, and that, subsequently, 5-HTP administration will improve task performance on these measures for individuals with high levels of ADHD traits.

## Materials and methods

### Participants

#### Participant recruitment.

Participants were recruited from the University of Sheffield’s volunteer database, community groups, and the University’s student population using posters, emails,advertisements, and websites such as the UK Adult ADHD Network. All recruitment materials included a link to the study information sheet and consent form for interested individuals, whereby they completed an online pre-screening survey. Eligible participants were then invited to book a 2.5-hour slot at the University of Sheffield to participate in the trial. Participants were compensated for their time with £30 Amazon vouchers or course credits for undergraduate psychology students.

No prior research is available measuring the impact of 5-HTP on distractibility, meaning that we were unable to use pre-existing data to inform power analysis and sample size. A priori power analysis for repeated measures ANOVA was completed using G-power [55], and calculated a participant number of 112 for a medium effect size (two-tailed, alpha = 0.05, beta = 0.08, effect size = 0.3). Recruitment stopped at 112 participants (89 female, mean age = 23.6 years) with usable data. An interim analysis was produced at 105 participants in preparation for the presentation of the data at a conference.

#### Inclusion criteria.

Participants between the ages of 18 and 65 were selected; the lower bound ensured only adults were assessed, and the upper bound to include a range of adults within working age in the UK, whilst maintaining a low chance of participants experiencing mild cognitive impairment [56]. Control participants were selected based on a score of 1 or less on the Adult ADHD self-report screener (ASRS v1.1, items 1–6) [57], whereas participants in the high ADHD group were selected based on a score of 4 or greater, considered highly consistent with ADHD. To broaden participation criteria to include nonclinical presentations of ADHD, participants in the high group were not required to have a clinical diagnosis of ADHD.

#### Exclusion criteria.

Participants were excluded following pre-screening if they were taking stimulant or non-stimulant medication for the treatment of ADHD to allow us to assess the effect of 5-HTP without the interference of psychostimulants. Individuals who used other serotonin-altering medications, such as selective serotonin reuptake inhibitors, were also excluded. Smokers or vapers were excluded due to the considerable psychoactive effects of nicotine. Other exclusion criteria included pregnant and breastfeeding individuals, and individuals with lactose intolerance or a dairy-free diet as placebo tablets contained lactose. Individuals with dyslexia were also excluded to minimise extraneous factors that may affect reaction time and accuracy on letter-presenting tasks.

### Trial design

**Fig 1:**
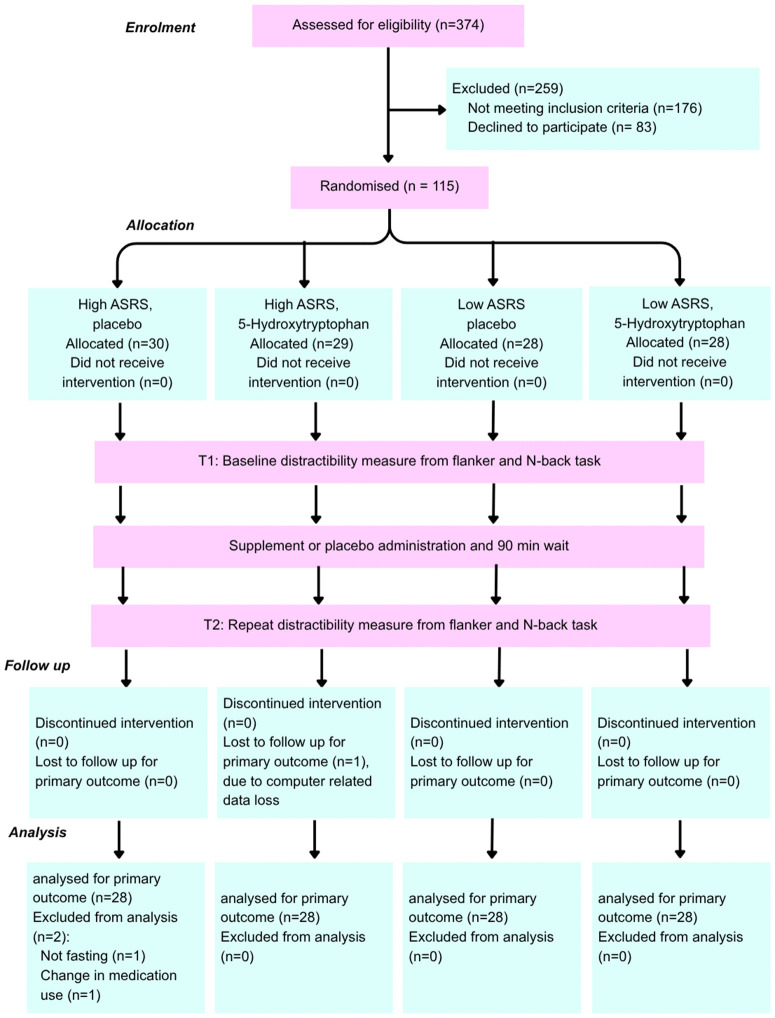
Study design (CONSORT) flow chart: Flow diagram outlining the study protocol, including participant recruitment, randomisation, allocation and analysis [58].

The study consisted of a randomised, parallel-group trial in a 2 (group: high ADHD traits vs low ADHD traits) x 2 (intervention: placebo or 5-HTP) x 2 (time: pre- and post-administration) design. Participants were stratified into two groups of high and low levels of ADHD traits using the ASRS, and assignment to either the acute, high-dose 5-HTP administration group or placebo group was randomised in a 1:1 allocation ratio. Both participants and trial investigators were blinded to the allocation. The trial primary outcome was a change in distractibility, measured by recording mean reaction time, standard deviation of reaction time, accuracy, and the percentage of false positives in two distractor tasks (See Fig 1). Analysis centred on observing differences between these values pre- and 90 minutes post-administration. Public involvement extended only to participation in the trial.

The study protocol and analysis plan were preregistered on the Open Science Framework (OSF) (https://doi.org/10.17605/OSF.IO/H6KET) and later registered with ISRCTN (reference ISRCTN12905294). No changes were made to the preregistered protocol. De-identified participant data and experiment code can be accessed at the University of Sheffield Online Research Data Repository (https://doi.org/10.15131/shef.data.29183702). Reporting aligns with CONSORT 2025 guidelines [58].

#### Ethics and harms.

The study was approved by the University Research Ethics at the University of Sheffield on the 4th of March 2024 (RN 058170). All participants had access to the information sheet and online consent form. Consent was taken twice: first for the collection of pre-screen data, and again before participation in the trial. Written consent was collected electronically. Participants were recruited between 20^th^ May 2024 and 27^th^ February 2025. No follow-up was conducted after the in-person session.

Harms were detailed and assessed in a risk assessment prior to initiation of the trial. 5-HTP has been widely established as safe for human consumption, with some common adverse effects. As such, adverse events were assessed non-systematically by collating reports from participants throughout the trial.

### Materials and measures

#### Online pre-screen and ASRS v1.1.

Potential participants were required to complete an online screening survey to assess whether they met the inclusion criteria. In the interest of brevity, participants were asked for minimal demographic information of age and sex assigned at birth. Participants were then asked screening questions to determine eligibility. If answers indicated exclusion, participants were screened out of the survey. Participants were also asked to indicate use of psychostimulants or serotonergic-intervening medications and if they had previously received a diagnosis of ADHD, autism, dyslexia or a mental health condition.

ADHD-like traits were assessed in the pre-screen using the 6-question ASRS v1.1 screener, a well-validated clinical tool used in screening for ADHD [57]. Participants answered questions regarding their behaviour over the preceding 6 months on a Likert rating scale with the answers never, rarely, sometimes, often or very often. Questions 1–3 score 1 point for answers of sometimes, often or very often; any other answer is given 0 points. The remaining questions score 1 point for often or very often and 0 points for other answers. A score of 4 or more is considered a score highly consistent with an ADHD diagnosis. Therefore, the high ADHD traits group was defined as participants with a score of 4 or more. Reports of ASRS scores in the USA have previously found a mean score of 2.0. Therefore, participants with a score of 1 or less were determined as the low ADHD traits group [59].

All pre-screener respondents also completed the extended ASRS [57], a further 12 questions, that, when coupled with the screener, give 9 questions on hyperactive-impulsive traits and 9 questions on inattentive traits. Questions 9, 12, 16, and 18 were scored in the same manner as questions 1–3, with all other questions scored with 1 point for answers of often or very often and 0 points for answers of never, rarely or sometimes. Answers were used to characterise the sample and better understand the presentation of ADHD subtypes within the high ASRS group.

The lead researcher reviewed pre-screen responses within 5 days of completion, and respondents were contacted via email. Eligible participants were then invited to book a laboratory testing slot.

#### 5-hydroxytryptophan and placebo preparations.

Participants in the intervention group were given a 200 mg dose of 5-HTP orally, based on a recent study looking at acute 5-hydroxytryptophan administration and social cognition [60]. 5-HTP was obtained from Nature’s Best supplements in a tablet containing 3982 mg of Griffonia seed extract, providing 100 mg of 5-hydroxytryptophan, along with calcium carbonate, anti-caking agents (silicon dioxide, stearic acid and magnesium stearate), and tablet coating (hydroxypropyl methylcellulose, glycerol). Participants were given 2 tablets with water. Placebo tablets were sucrose-lactose tablets obtained from Ainsworth’s homoeopathic remedies. Again, participants were given two tablets with water.

#### Task- relevant distractor: Flanker task.

Participants completed an adapted version of the Eriksen flanker test (sets of chevrons were presented rather than the letter sets presented in the original task) [61], considered a reliable test of response to task-relevant distractors. There is evidence that ADHD individuals experience increased reaction time and reduced accuracy on such tests compared to a typically developed population, and that ADHD symptom remission is linked to improved performance on the flanker test [62,63].

**Fig 2:**
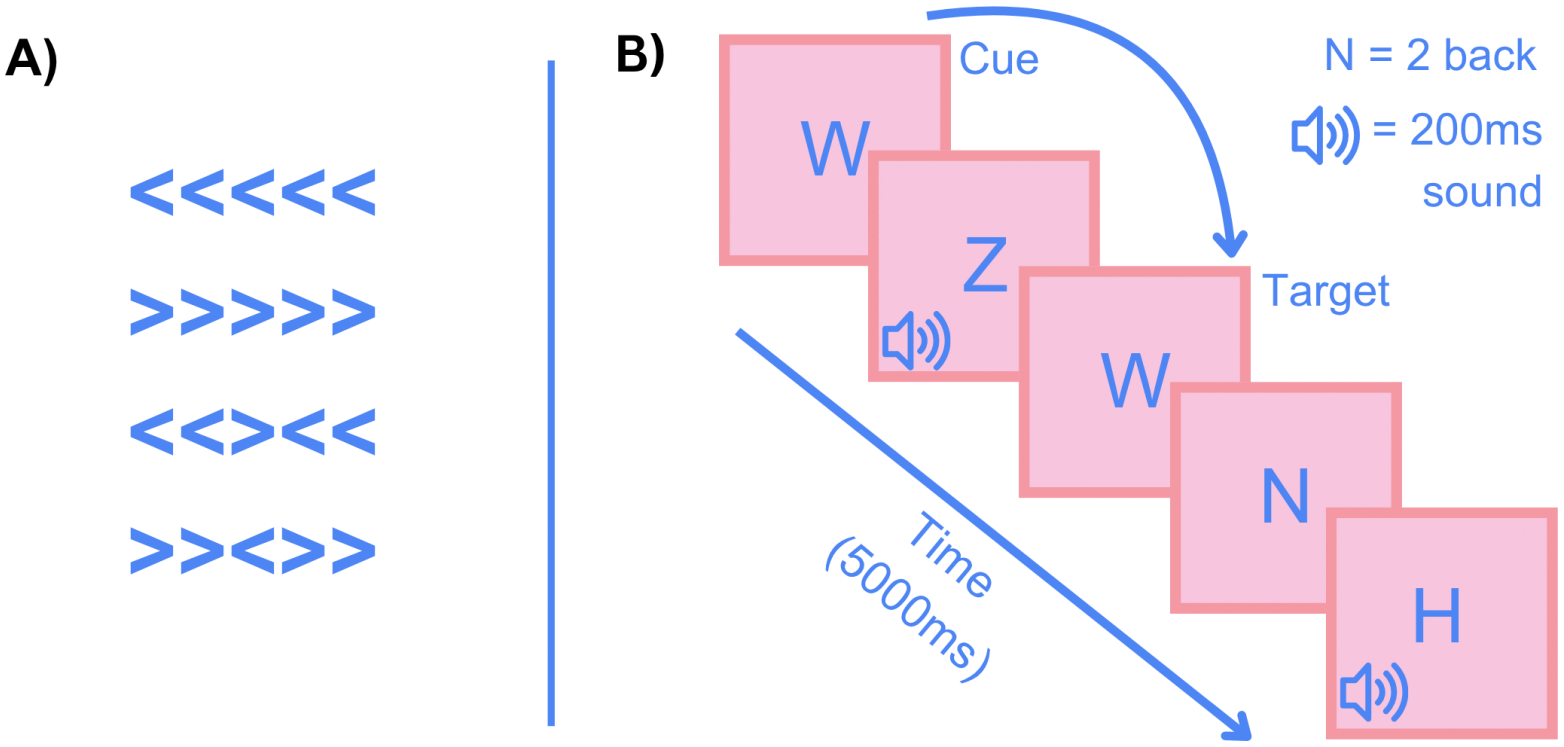
Stimuli used in the flanker and N-back tasks: A) the 4 stimuli, congruent and incongruent, displayed in the flanker task. **B)** Schematic of stimulus presentation in the n-back task.W forms a cue, and a target response, and auditory stimuli are present on non-cue, non-target letters.

The flanker task was built and delivered using Psychopy 2023 2.3 [64]. Participants were presented with a series of 5 chevrons and asked to report the direction of the middle chevron using a key press (Z for left, M for right). Chevrons were displayed in either congruent (>>>>>, <<<<<) or incongruent (<<><<,>><>>) displays (see Fig 2). The chevrons were presented for 200 ms, and following participant response, a fixation cross was presented for 1000 ms prior to the next trial. Participants completed a 20-trial practice block prior to the test phase. The testing phase consisted of 10, 40-trial blocks, each presenting each combination of chevrons 10 times in a random order, resulting in 400 trials with 100 presentations of each condition. Participants were given a minimum 30 s break between each block, with the task taking around 15 mins to complete. Participants’ performance was assessed with several measures: mean reaction time, standard deviation of reaction time, and accuracy as a percentage for congruent and incongruent trials.

#### Task- irrelevant distractor: N-back task.

Participants also completed a task-irrelevant distractor task. Studies looking at task-irrelevant distraction have observed inflated scores in reaction time in individuals with ADHD or increased ADHD traits [65,66]. Similar changes in response occur in oddball paradigm tasks, where novel auditory stimuli are presented [67,68]. As a result, we incorporated novel auditory stimuli as task-irrelevant distractors into a version of the N-back test. This decision to use an N-back test was based on evidence that individuals with ADHD have poorer performance on the N-back test compared to controls and that ADHD medications ameliorate the observed deficits. [69].

Participants were asked to fixate on a fixation cross for 600 ms, then presented with a letter (H, K, N, W, or Z, based on the letters from the Eriksen flanker for their similarity) [61]. Letters were presented for 400 ms, and a further 600 ms elapsed before the next trial began, giving a 1000-ms window for reaction times. Participants were asked to recall if the letter they were presented with matched the letter presented two trials ago, and to answer affirmatively by pressing the spacebar (see Fig 2). On 25% of trials, a 200 ms, 85 dB noise was presented simultaneously with a trial letter. Sounds were assigned using stratified randomisation, with sounds assigned to cues, target and neutral letters in a 1:1:3 ratio, meaning that a sound was played on 25% of each condition. The study consisted of a 25-trial practice block, one block without sound (classed as silent trials), and 4 blocks with auditory stimuli. Presentation order of the auditory and silent blocks was counterbalanced between participants in a 1:1 ratio. Each block lasted approximately 2 min 40 s, with a minimum 30 s break between blocks. The task took around 16 min to complete. Participants’ performance was measured using reaction time, standard deviation of reaction time, and accuracy as a percentage of targets hit and as a percentage of false positive responses.

#### Randomisation.

Multiple components were randomised throughout the recruitment and testing phases of the trial. Upon completion of the pre-screen survey, participants were assigned a number that would be used to identify their data for the rest of the study. Using a random number generator, randomisation was stratified by group (high ADHD traits and low ADHD traits) with a 1:1 allocation to intervention and placebo conditions, recorded as A and B. Task order and block presentation in the n-back task were counterbalanced following the same procedure. Randomisation was completed and implemented by the lead investigator. All randomisation was produced using the web applications available at http://random.org.

To blind investigators enrolling, assigning and delivering the protocol, an assistant extraneous to the trial placed doses of placebo and 5-HTP tablets in A or B envelopes. The contents of A and B were then written down and sealed in an envelope to be opened at the completion of the trial. Participants were also blind as to what intervention they received. Interventions were both tablets in the same quantity, with 5-hydroxytryptophan tablets being slightly larger and darker in colour than the placebo tablets; thus, they were concealed from the investigator using envelopes.

### Procedure

Following engagement with recruitment, individuals were required to complete the pre-screening survey, providing information on demographics and ADHD traits. Responses were reviewed, and eligible participants were contacted by email with a link to book a 2.5-h slot at the University of Sheffield for the trial. Participants were contacted 3 days prior to the session with further details and a reminder. Participants were asked at this time to complete a food diary for the 48 h preceding the test, logging food, alcohol and medication consumption. Participants were also asked to refrain from consuming alcohol, nicotine, or caffeine on the day of their appointment, and to refrain from eating for two hours before their appointment.

Upon arrival, participants were given an information sheet,asked to complete a consent form, and reminded that they may withdraw consent at any point during the study. Participants were seated in a quiet room with a monitor and a keyboard, with an investigator present. The investigator explained the tasks to participants and presented them in their previously assigned, randomised order. Baseline tasks took around 35 min to complete.

Following completion of the initial tasks, participants were given an envelope containing either 5-HTP or placebo and a glass of water and were observed swallowing the tablets. Participants were then shown to a separate room where they were allowed to participate in a self-chosen leisure activity whilst they waited. The waiting period was 90 ± 5 min, based on previous pharmacokinetic reports of 5-hydroxytryptophan levels in serum and cerebrospinal fluid [[70].

Once the 90 min wait period was completed, participants returned to the quiet room and completed the two tasks again in the exact manner as in the baseline test phase. Upon completion, participants were debriefed and provided with researcher contact details. The procedure took approximately 2 h and 40 min to complete per participant.

### Data preparation and statistical analysis

Individual data was prepared by removal of reaction times outside of the range 200–3000 ms for the flanker task and reaction times under 200 ms for the N-back task, based on mean population values of simple human reaction time, and boundaries suggested by the NIH toolkit for flanker analysis [71,72]. Values that fell outside of ±3 standard deviations of the individual’s mean were also removed, following recommendations for reducing outliers in reaction time data [73]. All participants maintained at least 95% of data for each task. No participants had missing or incomplete data. Mean and standard deviation of reaction time values were calculated, as well as accuracy as a percentage of correct hits. The percentage of false positives was also calculated for the N-back task.

All analyses were completed using IBM SPSS Statistics (Version 29) and, unless otherwise specified, included all participants and were performed with a two-tailed significance value of 5%. Data were checked for normality using histograms, measures of skew and kurtosis and the Kolmogorov-Smirnov normality test. Significant outliers were noted in all measures of the flanker task, and in the standard deviations of reaction time and false positives in the N-back task. As such, these values were winsored prior to analysis. Due to the abnormality of the distribution, Box-Cox transformation was used to normalise all measures of the flanker task and the standard deviation of reaction time on silent trials and of false positives in the N-back task. This normalisation still resulted in a non-normal distribution of congruent accuracy in the flanker task, but given the robustness of ANOVAs, parametric testing was still used in the analysis [74]. Descriptives statistics, including means, standard deviations (SD) and percentages, were used to characterise the data.

Data analysis sought to answer three key questions: did the N-back and Flanker tasks manipulate distractibility, did the two tasks discriminate between individuals with high and low ADHD traits, and finally, did 5-HTP supplementation affect performance on the tasks. This was assessed by looking at differences in reaction time, individual variance of reaction time (standard deviation), and task accuracy. The N-back additionally looked at the rate of false positive hits as a metric of distractibility.

Test efficacy was assessed using paired t-tests between control (silent [N-back] or congruent [flanker] trials) and distractor (auditory stimulus [N-back] or incongruent [flanker] trials) condition measures at the pre-administration timepoint. To compare task performance between ASRS groups, a MANOVA was used to observe all of the variables of the flanker task and the variables of the N-back task, to look for any gross group differences. A 2x2 split-plot ANOVA was used to assess differences between the ASRS group and distractor/no distractor conditions, and independent t-tests to look at differences between groups on each measure. Finally, Intervention outcomes were first assessed with a 2x2 ANOVA (intervention x timepoint) to identify effects of the 5-HTP regardless of ASRS group, and then further assessed using a 2 x (Group: High ADHD traits, low ADHD traits) 2 x (Intervention: 5-HTP, placebo) 2 x (time: pre and 90 min post) ANOVA. ANCOVAs with age and baseline ASRS score were also performed. A final ANOVA was conducted with participants experiencing adverse events removed, to assess the impact these events had on the outcome measures.

## Results

### Study population

In total, 374 participants completed the pre-screening survey, of which 176 were excluded for not meeting the eligibility criteria. The most common reason for exclusion was a non-eligible ASRS score (N = 83), although a considerable number of participants were excluded due to the use of serotonin-affecting medications (N = 37), smoking status (N = 25), dietary needs (N = 19) and a diagnosis of dyslexia (N = 12). A further 83 participants passively withdrew from the study prior to assignment to a trial group by not following up. 3 participants’ data were excluded from the analysis prior to the completion of data collection; one participant had a change to their medication status, one did not follow the fasting requirements, and one was excluded due to a computer error in the laboratory.

Demographic and clinical characteristics of each group are described in Table 1. The groups did not significantly differ in age, gender, or clinical diagnoses when looking between groups of the same ASRS status. There was a significant difference in ASRS scores in each measure between high and low ADHD trait groups, but additionally, there was a significant difference between high ADHD trait groups on the screener score and the hyperactive component – in both instances, the intervention group had a higher score (Table 2). Given the pronounced differences between low and high groups, this was not a concern.

**Table 1:** Demographic characteristics of participants. No significant difference was observed between groups for age (F = 1.89, p = 0.132) or gender ratio (F = 1.28, p = 0.283).

Variables	High ASRS score		Low ASRS score	
	Placebo	5-HTP	Placebo	5HTP
N	28	28	28	28
Age (years) M (SD)	24.60 (10.23)	21.32 (4.81)	24.40 (10.14)	24.32 (7.99)
Assigned female at birth N (%)	21 (75)	23 (82.14)	24 (85.71)	21 (75)
Gender undisclosed N (%)	1 (3.57)	0	0	0
Diagnosed with ADHD N (%)	4 (14.29)	4 (14.29)	0	0
Diagnosed with Autism N (%)	2 (7.14)	3 (10.71)	0	1 (3.57)
Diagnosed with anxiety or depression N (%)	4 (14.29)	3 (10.71)	2 (7.14)	1 (3.57)
Diagnosed with Autism and ADHD N (%)	1 (3.57)	0	0	0

**Table 2:** Scores on the ASRS screener, hyperactive and inattentive subsections, and subsequent subtypes of the high ASRS group.

	High ASRS score		Low ASRS score		F	Significance
	Placebo	5-HTP	Placebo	5HTP		
ASRS screener score M (SD)	4.75 (0.79)	5.11 (0.78)	0.54 (0.50)	0.61 (0.49)	478.12	**<0.001**
Inattentive score M (SD)	7.07 (1.40)	7.46 (1.19)	1.25 (1.03)	1.21 (1.12)	822.3	**<0.001**
Hyperactive score M (SD)	4.78 (2.10)	5.64 (1.93)	0.93 (0.90)	1.01 (1.01)	10.38	**<0.001**
Combined type N (%)	13 (46.43)	22 (78.57)	0	0		
Inattentive type N (%)	13 (46.43)	6 (21.43)	0	0		
Hyperactive type N (%)	1 (3.57)	0	0	0		
Subclinical scorers N (%)	1 (3.57)	0	28 (100)	28 (100)		

There was an unequal distribution of ADHD traits, with 62.5% (N = 35) had scores suggesting combined-type traits, 33.9% (N = 19) with scores suggesting predominantly inattentive traits, and 1.8% (N = 1) with predominantly hyperactive traits. A further 1.8% (N = 1) had a screener score within inclusion criteria, but with the extended ASRS did not reach clinical scores in either domain.

### Did the N-back and Flanker tasks manipulate distractibility?

Paired t-tests between control and distractor stimuli revealed no significant difference between the distractor and non-distractor conditions for the accuracy, reaction time or standard deviation of the reaction time in the N-back task at time point 1 (see S1 File). However, there was a significant difference between the number of false positives on distractor and non-distractor trials, although surprisingly, the silent trials (M = 42.72%, SD = 16.54%) had a significantly higher number of false positives than the auditory trials (M = 39.01%, SD = 15.84%), t (111) = −3.08, p = 0.003.

All measures of the flanker task differed significantly between the two conditions; incongruent trials (M = 478.66ms, SD = 68.28ms) had a longer reaction time than congruent trials (M = 425.12ms, SD = 57.51ms) (t(111) = 22.35, p < 0.001), and incongruent trials had a lower accuracy (M = 88.95%, SD = 7.84%) than congruent (M = 98.01%, SD = 3.81%) (t(111)= −13.845, p < 0.001). There was also a significant difference in the mean standard deviation of reaction times for incongruent and congruent trials, such that reaction times on incongruent trials (M = 109.08ms, SD = 53.4ms) varied more widely than reaction times on congruent trials (M = 92.41ms, SD = 41.06ms; t(111) = −13.845, p < 0.001). It was thus determined that the presentation of auditory stimulus in the N-back did not provide a significant distractor effect. However, the flanker task provided a significant distraction that impacted reaction time, accuracy and standard deviation of reaction time.

### Did the two tasks discriminate between individuals with high and low levels of ADHD traits?

#### Initial assessment.

When considering the difference in performance between the ADHD trait groups, MANOVA revealed a significant effect of ADHD trait group for the combined N-back variables F(8,103) = 2.156, p = 0.037; Wilks λ = 0.857. Further, analysis with 2 x 2 split-plot ANOVA between ADHD trait group and distractor condition demonstrated a main effect of ADHD trait group and the standard deviation of reaction time (F(1,110) = 5.20, p = 0.025, ηp^2^ = 0.045). Interestingly, between silent and auditory conditions, the standard deviation increased for the low ADHD trait group (silent M = 155.14ms, SD = 30.41, audio M = 165.05ms, SD = 17.14), whereas it decreased for the high ADHD trait group (silent M = 174.96ms, SD = 25.75, audio = 171.85ms, SD = 20.2). No significant differences were observed in accuracy, false positives or reaction time (see S2 and S3 File). When looking at the effect of the ADHD trait group on univariate measures, significant differences were observed in the standard deviation of reaction time in the N-back on silent trials (Low (M = 155.06, SD = 27.80), High (174.34, SD = 29.74), t(110) = 3.723, p < 0.001). Standard deviation in reaction time was approaching significance in the auditory trials (Low (M = 165.05, SD = 20.20), High (M = 171.85, SD = 17.14), t(110) = 1.919, p = 0.058). The N-back results highlight some differences in performance between high and low ASRS groups, chiefly that individuals with high levels of ADHD-like traits have a greater standard deviation of reaction time in the absence of an auditory stimulus.

The ADHD trait group had a non-significant effect on accuracy, reaction time and standard deviation of the reaction time of the flanker task in both multivariate or univariate analyses (see S4 and S5 File). As such, it was determined that the flanker did not discriminate between high and low ADHD trait groups.

### Effect of 5-hydroxytryptophan

**Fig 3:**
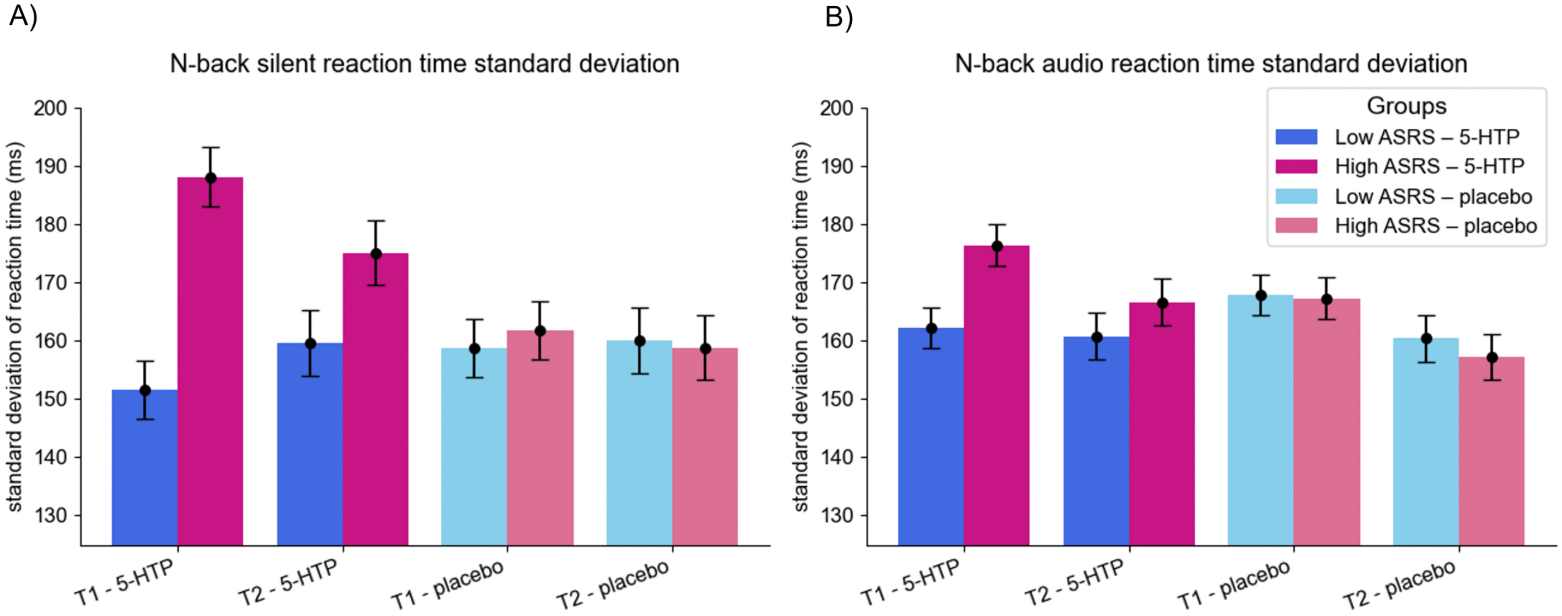
N-back performance across ASRS groups, conditions and timepoints. Comparison of mean scores in measures of standard deviation of reaction time across the N-back, split into intervention and ADHD trait groups at both pre- and post-administration (blue being low ADHD traits, pink being high ADHD traits. Darker shades are 5-HTP, lighter placebo). Error bars represent the standard error of the mean. **A)** Standard deviation of the reaction time in silent N-back trials. **B)** Standard deviation of the reaction time in auditory N-back trials.

Prior to analysing the effect of 5-HTP, analysis was completed to ensure that differences observed in standard deviations of reaction times and false positives between the high and low ADHD trait groups pre-administration in the N-back task remained when split down into the four intervention groups. ANOVA between the four groups highlighted a significant main effect of group on non-distractor standard deviation of the reaction time in the N-back (F(3,108) = 10.03, p < 0.001), and post-hoc analysis with Tukey correction identified significant differences between the high ADHD trait 5-HTP group and all other groups (Table 3). A significant main effect of group was also found on the standard deviation of the reaction time in auditory trials (F(3,108) = 2.837, p = 0.042), although the difference appeared to be only between the high and low 5-HTP groups (Table 3, Fig 3). Significance was also observed in the percentage of false positives in silent trials (F(3,108) = 3.675, p = 0.014) and the percentage of false positives in auditory trials (F(3,108) = 3.745, p = 0.013), but post-hoc analysis identified differences within the ADHD trait groups rather than between them (Table 3).

**Table 3:** Between-group difference results from Tukey post-hoc analysis of one-way ANOVA looking at effect of intervention group on N-back performance measures at time point 1.

Measure	Group 1 (n = 28)	Group 2 (n = 28)	Mean difference	Significance	95% confidence interval	
					Lower	Upper
Standard deviation of reaction time non-distractor	High placebo	High 5-HTP	−26.32	**.002**	−44.93	−7.71
	High placebo	Low placebo	3.07	.973	−15.54	21.68
	High placebo	Low 5-HTP	10.26	.478	−8.35	28.87
	High 5-HTP	Low placebo	29.38	**<.001**	10.77	47.99
	High 5-HTP	Low 5-HTP	36.58	**<.001**	17.97	55.19
	Low placebo	Low 5-HTP	7.20	.744	−11.41	25.81
Standard deviation of reaction time distractor	High placebo	High 5-HTP	−9.12	.259	−22.02	3.78
	High placebo	Low placebo	−0.624	.999	−13.53	12.28
	High placebo	Low 5-HTP	5.09	.733	−7.81	17.99
	High 5-HTP	Low placebo	8.50	.319	−4.41	21.40
	High 5-HTP	Low 5-HTP	14.21	**.025**	1.31	27.11
	Low placebo	Low 5-HTP	5.71	.656	−7.19	18.62
false positives non-distractor	High placebo	High 5-HTP	−13.50	**.011**	−24.64	−2.36
	High placebo	Low placebo	−3.64	.829	−14.78	7.50
	High placebo	Low 5-HTP	−3.75	.816	−14.89	7.39
	High 5-HTP	Low placebo	9.86	.102	−1.28	21.00
	High 5-HTP	Low 5-HTP	9.75	.108	−1.39	20.89
	Low placebo	Low 5-HTP	−0.11	1.0	−11.25	11.03
false positives distractor	High placebo	High 5-HTP	−12.90	**.007**	−23.16	−2.64
	High placebo	Low placebo	−7.31	.252	−17.56	2.95
	High placebo	Low 5-HTP	−4.70	.630	−14.96	5.55
	High 5-HTP	Low placebo	5.59	.488	−4.664	15.85
	High 5-HTP	Low 5-HTP	8.20	.165	−2.06	18.45
	Low placebo	Low 5-HTP	2.60	.911	−7.65	12.86

As ADHD trait group still had a significant effect on standard deviation of reaction time in N-back task measures when split into intervention groups, a 2 x 2 x 2 ANOVA between ADHD trait group, intervention and time point was used to assess if 5-HTP administration impacted the initial differences observed between groups. There was no 2-way interaction between 5-HTP administration and time point on the standard deviation of reaction times in the N-back task (see S4 Table). There was however, a difference between silent trial accuracy in the N-back between 5-HTP and placebo at pre- and post-administration measures F(1,110) = 6.693, p = 0.011, ηp2 = 0.057). Placebo saw an increase in mean from 53.25% to 60.82% of silent targets hit, whereas 5-hydroxytryptophan administration had an initial mean of 54.08%, which was reduced to 53.69% post-administration. No significant 3-way interactions were observed (see S5 Table).

To check that effects, or lack of, were not due to covariance, ANCOVAs were conducted. Age and baseline ASRS score did not provide any different results to the initial ANOVA when controlled for as covariates (see S6 and S7 Table).

### Adverse events

Several participants (N = 11) reported adverse events during the experiment, including nausea (N = 2), vomiting (N = 4) and significant fatigue (N = 5). All adverse event reports were from participants on the 5-HTP intervention, and all participants who reported nausea or vomiting were female. Otherwise, there appeared to be no unifying characteristic linked to the experience of adverse events. Some participants (N = 4) also reported significant hunger, which they believed may impact their task performance; all but one of these participants were on the trial’s placebo arm.

To explore the mediation of these effects, a further ANOVA excluding participants who experienced adverse events was completed. Similarly to the initial three-way ANOVA, a difference between silent trial accuracy in the N-back was observed (F(1.93) = 5.636, p = 0.20, ηp2 = 0.057), whereby participants in the placebo group saw an improvement in accuracy (52.5% to 60.5%), and the 5-HTP group saw no change (51.1% to 52.2%). No other two or three-way interactions were observed.

### Summary of findings

As initially established, the analysis of this study sought to answer three core questions: do the tasks manipulate distraction, do the tasks discriminate between high and low levels of ADHD traits, and does 5-HTP administration affect distractibility. The flanker task was found to manipulate distraction with significant differences between congruent and incongruent trials. The N-back with auditory stimulus did not provide a distractor effect.

When examining ADHD trait discrimination, the N-back task highlighted differences between high and low ADHD trait groups, in both overall task performance and in the standard deviation of reaction times. No difference in performance was found in the flanker tasks.

Evaluation of the impact of 5-HTP administration found one difference between intervention and placebo groups pre- and post-intervention, where placebo accuracy in the N-back increased between time points but decreased for 5-HTP. No other differences were noted because of 5-HTP administration, and there was no significant interaction related to ADHD trait groups. Analysis of the effect of adverse events on potential outcomes yielded no change to these findings.

### Discussion

This study aimed to assess the effects of acute 5-HTP administration on the core ADHD symptom of distractibility in individuals with high levels of ADHD traits. We hypothesised that 5-HTP administration would reduce distractibility in individuals with high levels of ADHD traits, with a group of individuals with low levels of ADHD traits present to act as a control group. The data highlighted no significant improvements due to 5-HTP on any measure of distractibility in either group. Currently, no literature has examined the impact of 5-HTP administration in the management of ADHD traits; thus, the results, despite not indicating an effect of 5-HTP administration, are novel. Given that the tasks did not effectively discriminate between high and low ADHD traits, and a lack of distractor effect from the N-back task, it is difficult to draw conclusions about 5-HTP impact on ADHD traits.

### Effectiveness of the flanker task

In the flanker task, congruency had a significant effect on all measures, indicating that the distractor condition was indeed distracting, as has been widely observed previously [75,76]. As such, the flanker task provided results similar to those observed in previous work, whereby incongruent trials provide a distraction that impacts task performance. However, the flanker failed to highlight a difference between groups with high and low levels of ADHD traits.

A previous systematic review of flanker performance found that individuals with ADHD generally had slower reaction times and greater error rates, but, as with much work in ADHD, it focused on a child population [63]. Given that attentional networks mature and develop over time, and that performance in tasks such as the flanker develops and differs between childhood and adulthood, it is perhaps unsurprising that the same results are not mirrored in adult populations [77]. There are fewer instances of the flanker task in ADHD adults, but results are conflicting; one study reports individuals with ADHD as having a longer reaction time and a higher level of accuracy, another found no difference in reaction time and a lower level of accuracy, and another found differences in reaction time and standard deviation of reaction time but no differences in accuracy [78–80]. It is also possible that a lack of observed difference is due to the non-clinical sample used within this study.

It is also of note that there were several methodological differences between the flanker tasks used; Merkt et al. (2013) [78] used a paradigm where participants reported if a number was odd or even, and the distractors were either odd or even numbers, McLoughlin et al (2009) [80] presented two flanker arrows 100 ms before the initial paradigm, and Marquardt et al (2018) [79] presented the flanker for 100 ms, rather than the 200 ms used in this protocol. All these features may make the task more challenging and may have better highlighted differences between groups; the current study had similar accuracy and congruent reaction times to those seen in Marquardt’s (2018) [79] work, but a considerably lower incongruent reaction time, and considerably lower reaction times and higher accuracies than those observed in Merkt et al., (2013) [78]. As such, it is possible that results from the current study skew lower due to the relative ease of the task.

### Effectiveness of the N-back task

With regards to the N-back task, the presence of the auditory stimulus did not create a distractor effect. The task did, however, show some discrimination between high and low ADHD traits groups, whereby individuals with higher levels of ADHD traits had a greater standard deviation of reaction time in the silent condition, and saw a decrease in standard deviation in the presence of an auditory stimulus. The low ADHD traits group instead saw an increase in the standard deviation of reaction time with an auditory stimulus present.

There are several possible reasons as to why the N-back task failed to distract. Previous work with unrelated auditory stimuli in a visual task has demonstrated a distraction effect, but there has been a focus on the novelty of auditory stimuli presented in comparison to the presentation of a standardised sound, whereas the current study used only a standardised beep compared to silence [67,68]. However, other work using novel auditory stimuli has also observed alerting or orienting effects, whereby performance improved with the presence of novel auditory stimuli [81,82]. There are several theories for such variance in performance; firstly, that working memory load may modulate response to novel sounds, where lower working memory load results in greater distraction due to sound, thus the cognitive load of the N-back task may have contributed to a lack of distraction effect [81]. Alternatively, the informational value of the sound, i.e., whether it predicts a target response, may also modulate whether a sound is distracting or not in experimental conditions [83]. With regards to the current study, it is possible that the repeated use of the same auditory stimulus reduced its effect as a distractor, although given that there appears to be no unifying trend in performance changes over time, this may not be the case. Furthermore, the volume of the sound may have played a role in the N-back’s failure; distraction by auditory stimuli has been considered to have a similar effect when at different volumes within the range of typical human speech, but there is some suggestion that at loud levels (above 80dB), the sound may present an alerting effect [84]. Again, evidence here is conflicting, and some work has found increased reaction time with increased volume [85]. As such, it is unclear as to why the N-back task failed to distract, but differences observed in the N-back will instead be considered primarily as differences in working memory.

### ASRS scores and task performances

The one difference observed between ADHD trait groups was the standard deviation of reaction time in the N-back task, whereby individuals with high levels of ADHD traits scores had a higher standard deviation than those with a low score. Response variability has been observed to differ between ADHD and neurotypical groups in several reports [86,87]. Such differences are postulated to be because of occasional lapses of attention and may often result in a larger magnitude difference than other neuropsychological markers, such as reaction time or accuracy in tasks [88]. Such changes have also been paired with the assessment of stimulant medication, whereby the administration of stimulants reduces response variability [87,88].

### Efficacy of 5-hydroxytryptophan

5-HTP administration only resulted in one effect, whereby individuals on placebo saw an improvement in the accuracy on the silent portion of the N-back task, and individuals taking 5-HTP saw no real change to their performance. This lack of other changes could indicate that 5-HTP did not reduce distractibility or affect working memory, but further considerations should also be made. Aside from the significant caveats that the flanker did not provide discrimination between the ADHD trait groups and the N-back failed to work as a distractor, the absence of changes between pre- and post-administration could also be related to the dose of 5-HTP used. A 200 mg dose was used based on previous research where a single 200 mg administration resulted in changes in behaviour on moral judgement tasks [60]. Across the literature, dosage of 5-HTP varies greatly, with some individuals using 5-HTP for myoclonus taking single doses of up to 725 mg, but within the literature for depression, doses from 50–300 mg are standard and result in symptom improvement [70,89]. With regards to 5-HTP and attention, preclinical work in macaques used a dose of 20 mg/kg, significantly higher than the dose used here [51].

Alongside dose, dose frequency should also be considered; it is well accepted that other serotonergic medications, such as selective serotonin reuptake inhibitors, require long periods of use before providing symptomatic relief, potentially because of the inhibitory activity of the 5-HT_1A_ receptor, which reduces the activity of serotonergic neurons in the initial increase of serotonin [90]. Such a possibility should also be considered with 5-HTP, where a longer administration may be needed to provide benefits. Furthermore, the dose given does not directly reflect central bioavailability; around 95% of serotonin synthesis occurs in the gut, and it is likely that a significant portion of the 5-HTP was indeed metabolised in the periphery [91]. It is possible that a higher dosage than 200 mg is needed to observe changes in performance on cognitive tasks.

Although direct measures of central serotonin were not used, the profile of adverse effects reported provides some indication that central serotonin likely increased. Firstly, the reporting of fatigue in several participants is promising; 5-HTP has been widely observed to improve sleep, and is believed to occur via synthesis of melatonin, one of the end metabolites in serotonin synthesis [54,92]. Additionally, presentation of nausea and vomiting also indicate that the dosage was significant enough to induce adverse events; however, some of this may be linked to activation of 5-HT_3_ receptors in the gastrointestinal tract, which would be an indicator of raised peripheral serotonin rather than central changes [91]. From analysis, it appears that the adverse effects did not contribute to the null findings of this study, but as adverse events were dependent on self-report, it is possible that not all participants who experienced fatigue, which could affect reaction times and accuracy, reported it. As ADHD is a biologically and clinically heterogeneous disorder, it is also possible that not every individual with high levels of ADHD traits presents with the same reduction in serotonin, nor the same genetic profile, which would result in reduced synthesis. For example, TPH2 variants, which have been linked to reduced 5-HTP levels, have been linked to ADHD in some cohorts, but not in others [20,34,93]. As such, future work should consider looking at baseline levels of serotonin and its subsequent metabolites, as well as genotyping, to better understand variance within the sample and characterise responses.

One other possible reason for the lack of differences between groups could be the use of a pre-clinical population. ASRS screening has been reported to have a high validity, with a classification accuracy of over 90%; thus, it was deemed appropriate for determining ASRS groups [57]. Comparison of ASRS scores between individuals who reported an ADHD diagnosis and others in the high ADHD traits group revealed a significant difference in hyperactivity, with ADHD diagnosed individuals having a higher mean score (6.625 compared to 4.915), but no differences were found in other ASRS scores (see S8 Table). As such, it appears that symptom severity, as noted by the ASRS screener, is not directly linked to diagnosis within the study cohort. As diagnosis was self-reported and not clinician-assessed, there is also room for error within this measurement. Future work should consider the use of clinical evaluation in determining the ADHD cohort, alongside potentially including collateral reports of trait presentation, as would be done in diagnosis.

### Study strengths

The current study, to our knowledge, is the first to assess the impact of 5-HTP supplementation on ADHD traits and has considerable strengths. Firstly, the study employed a randomised, controlled protocol with blinding, widely considered the gold standard for efficacy research [94]. The trial administrator blinding was maintained for 105 participants (93.75%); the last 7 participants were tested without trial administrator blinding as data needed to be prepared for presentation. Furthermore, the study population was overwhelmingly female (79% of the total study population), a group that has been historically underrepresented in ADHD diagnosis, treatment and research, addressing calls for greater female inclusion in ADHD research [95]. The study used well-validated measures of ADHD traits and tasks that had previously established differences between ADHD and typical cohorts [57]. Lastly, the dose of 5-HTP was selected based on evidence from previous research, and medication-naïve individuals were used to best isolate the effects of the intervention [60]. As a preliminary study, there is significant scope to extend and better understand the role and action of 5-HTP supplementation in ADHD, particularly in using tasks which are more sensitive to ADHD traits. One significant caveat with the study lies in the efficacy of the distractor tasks.

### Limitations

Although methodologically strong, the study has limitations. Firstly, although sample size recruitment was met and is the largest (and only) study assessing 5-HTP impact, the sample size was determined on power calculations for a medium effect size. As such, smaller effects may not have been possible to elucidate in the current sample. The sample also used a pre-clinical group. This could be considered a benefit, given it is well accepted that ADHD is underdiagnosed in the adult population, particularly in women, meaning the sample includes those typically under-represented in the current literature [59,95,96]. However, the ASRS does not equate to the depth of a clinical assessment. Regarding the intervention, there are several further limitations. Due to the small size of the study team, it was not possible to use central randomisation or outsource statistical analysis to individuals not involved in data collection, which could have resulted in less than optimal randomisation and blinding. There was also not a fully pre-defined statistical analysis plan, due to the anticipation of novel findings which may require exploratory analysis.

The most considerable limitation of the study is the efficacy of the cognitive tasks. The flanker task served as a distractor but did not discriminate between high and low levels of ADHD traits, so no conclusions about 5-HTP efficacy could be drawn from this. The N-back task showed the converse: no distractor effect was observed, but there was some discrimination between high and low ADHD traits, suggesting that conclusions about distractibility cannot be drawn from N-back results. Future work should consider tasks more sensitive to both ADHD traits and distractibility.

The current study did not employ direct measures of 5-HTP, serotonin, or any metabolites, meaning it is unclear as to whether serotonin was indeed increased from administration. Use of a fixed dose and acute administration also limits generalisability, as effects may vary in a dose-dependent manner. Crucially, it is well established that existing serotonergic therapies, such as Selective Serotonin Reuptake Inhibitors, require chronic administration to see clinical effects, even if serotonin itself increases rapidly. As such, a null result may be due to an acute intervention, and longer administration periods should be assessed in future work. Finally, when assessing the impact of supplementation, the current study focused on distractibility, which means that current findings cannot be generalised to other ADHD trait domains of hyperactivity and impulsivity.

### Conclusions

In conclusion, this is the first randomised, controlled trial looking at the effect of 5-HTP administration on ADHD traits. This preliminary study assessed the efficacy of acute dosage on distractibility in individuals with high levels of ADHD traits, with a low-level trait group as a control. The study found no significant effect of 5-HTP on performance in measures of distractibility or working memory, although this could be linked to the tasks not being sufficiently sensitive to ADHD traits, and the N-back task failing to work as a distractor. Further work is needed to establish the effect of 5-HTP on the other ADHD trait domains of hyperactivity and impulsivity, alongside observation of a range of doses to better elucidate the relationship between 5-HTP supplementation, changes to serotonin, and ADHD traits.

## Supporting information

S1 File N-back performance measures in distractor and non-distractor conditions at time point 1. (DOCX)

S2 File N-back performance measures split by ASRS group at time point 1 with univariate statistics. (DOCX)

S3 File N-back performance measures split by ASRS group at time point 1 with multivariate statistics. (DOCX)

S4 File Flanker performance measures split by ASRS group at time point 1 with univariate statistics. (DOCX)

S5 File Flanker performance measures split by ASRS group at time point 1 with multivariate statistics. (DOCX)

S1 Table N-back performance measures for the participants with ADHD diagnosis and lowest ASRS scorers matched group. (DOCX)

S2 Table Flanker performance measures for the participants with ADHD diagnosis and lowest ASRS scorers matched group. (DOCX)

S3 Table Mean performance across measures in the flanker and N-back task, split by ADHD subtype. (DOCX)

S4 Table ANOVA results for intervention x timepoint on performance measures in the N-back task. (DOCX)

S5 Table ANOVA results for intervention x ASRS group x timepoint on performance measures in the N-back task. (DOCX)

S6 Table ANCOVA results for intervention x ASRS group x timepoint on performance measures in the N-back task, age as covariate. (DOCX)

S7 Table ANCOVA results for intervention x ASRS group x timepoint on performance measures in the N-back task, Baseline ASRS score as covariate. (DOCX)

S8 Table ASRS scores of high ASRS group split by diagnosis status. (DOCX)

S1 Supplement Completed CONSORT checklist. (DOCX)

S2 Supplement Protocol as approved by ethical committee. (DOCX)
