# Supplementary material for: The effect of 5-hydroxytryptophan, a serotonin precursor, on adults with high levels of Attention Deficit Hyperactivity Disorder traits: A randomised, controlled trial
Source: PLoS One. 2026 May 20;21(5):e0349512. doi: 10.1371/journal.pone.0349512 (PMC13189352; doi:10.1371/journal.pone.0349512)
Supplement: S1 File — (DOCX) [file pone.0349512.s001.docx]

# Supporting information:

**S1: N-back performance measures in distractor and non-distractor conditions at time point 1.**

| Measure | Distractor (audio)  M (SD) | non-distractor (silent) M (SD) | t | p | Cohen's d |
| --- | --- | --- | --- | --- | --- |
| Accuracy | 51.54 (14.38) | 53.29 (17.00) | 1.754 | .082 | .166 |
| percentage of false positives | 39.01 (15.84) | 42.72 (16.54) | 3.084 | **.003** | .291 |
| reaction time (ms) | 589.56 (69.73) | 595.63 (96.91) | 0.981 | .329 | .093 |
| standard deviation of reaction time (ms) | 168.45 (18.96) | 164.70 (30.25) | 1.167 | .246 | .11 |
