# Supplementary material for: The effect of 5-hydroxytryptophan, a serotonin precursor, on adults with high levels of Attention Deficit Hyperactivity Disorder traits: A randomised, controlled trial
Source: PLoS One. 2026 May 20;21(5):e0349512. doi: 10.1371/journal.pone.0349512 (PMC13189352; doi:10.1371/journal.pone.0349512)
Supplement: S2 File — (DOCX) [file pone.0349512.s002.docx]

# Supporting information:

**S2: N-back performance measures split by ASRS group at time point 1 with univariate statistics.**

| Measure | Condition | High ASRS group | Low ASRS group | t | p | Cohen’s d |
| --- | --- | --- | --- | --- | --- | --- |
| Accuracy | Distractor | 51.11 (15.65) | 51.98 (13.12) | 0.321 | .749 | .061 |
|  | Non-distractor | 51.07 (17.02) | 55.5 (16.84) | 0.709 | .204 | .241 |
| percentage of false positives | Distractor | 39.41 (14.68) | 38.96 (15.92) | 0.154 | .878 | .029 |
|  | Non-distractor | 44.25 (15.51) | 41.19 (17.52) | 0.977 | .331 | .185 |
| reaction time in ms | Distractor | 591.40 (63.66) | 587.73 (75.85) | 0.277 | .782 | .052 |
|  | Non-distractor | 606.04 (83.38) | 585.23 (108.54) | 1.138 | .258 | .215 |
| standard deviation of reaction time in ms | Distractor | 171.85 (20.2) | 165.05 (17.14) | 1.919 | .058 | .363 |
|  | Non- distractor | 174.96 (25.75) | 155.14 (30.41) | 3.723 | **<.001** | .704 |
