# Supplementary material for: The effect of 5-hydroxytryptophan, a serotonin precursor, on adults with high levels of Attention Deficit Hyperactivity Disorder traits: A randomised, controlled trial
Source: PLoS One. 2026 May 20;21(5):e0349512. doi: 10.1371/journal.pone.0349512 (PMC13189352; doi:10.1371/journal.pone.0349512)
Supplement: S3 File — (DOCX) [file pone.0349512.s003.docx]

# Supporting information:

**S3:** **N-back performance measures split by ASRS group at time point 1 with multivariate statistics.**

| Measure | Condition | High ASRS group | Low ASRS group | F | p | ηp^2^ |
| --- | --- | --- | --- | --- | --- | --- |
| accuracy | Distractor | 51.11 (15.65) | 51.98 (13.12) | 1.905 | .17 | .017 |
|  | Non-distractor | 51.07 (17.02) | 55.5 (16.84) |  |  |  |
| percentage of false positives | Distractor | 39.41 (14.68) | 38.96 (15.92) | 1.295 | .258 | .012 |
|  | Non-distractor | 44.25 (15.51) | 41.19 (17.52) |  |  |  |
| reaction time in ms | Distractor | 591.40 (63.66) | 587.73 (75.85) | 1.936 | .167 | .017 |
|  | Non-distractor | 606.04 (83.38) | 585.23 (108.54) |  |  |  |
| standard deviation of reaction time in ms | Distractor | 171.85 (20.2) | 165.05 (17.14) | 5.199 | **.025** | .045 |
|  | Non- distractor | 174.96 (25.75) | 155.14 (30.41) |  |  |  |
