# Supplementary material for: The effect of 5-hydroxytryptophan, a serotonin precursor, on adults with high levels of Attention Deficit Hyperactivity Disorder traits: A randomised, controlled trial
Source: PLoS One. 2026 May 20;21(5):e0349512. doi: 10.1371/journal.pone.0349512 (PMC13189352; doi:10.1371/journal.pone.0349512)
Supplement: S4 File — (DOCX) [file pone.0349512.s004.docx]

# Supporting information:

**S4: Flanker performance measures split by ASRS group at time point 1 with univariate statistics.**

| Measure | Condition | High ASRS group | Low ASRS group | t | p | Cohen’s d |
| --- | --- | --- | --- | --- | --- | --- |
| accuracy | incongruent | 88.47 (7.19) | 89.49 (8.0) | 0.712 | .478 | .135 |
|  | congruent | 97.93 (1.71) | 98.05 (1.97) | 0.351 | .726 | .066 |
| reaction time (ms) | incongruent | 478.69 (58.94) | 481.12 (70.95) | 0.197 | .845 | .037 |
|  | congruent | 423.34 (52.49) | 427.44 (56.86) | 0.396 | .693 | .075 |
| standard deviation of reaction time (ms) | incongruent | 113.29 (52.95) | 106.26 (45.15) | 0.755 | .452 | 0.143 |
|  | congruent | 95.19 (40.22) | 91.33 (34.26) | 0.547 | .586 | 0.103 |
