# Supplementary material for: The effect of 5-hydroxytryptophan, a serotonin precursor, on adults with high levels of Attention Deficit Hyperactivity Disorder traits: A randomised, controlled trial
Source: PLoS One. 2026 May 20;21(5):e0349512. doi: 10.1371/journal.pone.0349512 (PMC13189352; doi:10.1371/journal.pone.0349512)
Supplement: S1 Table — (DOCX) [file pone.0349512.s006.docx]

# Supporting information:

**Table S6: N-back performance measures for the participants with ADHD diagnosis and lowest ASRS scorers matched group.**

| Measure | Condition | ADHD group | Low ASRS group | t | p | Cohen’s d |
| --- | --- | --- | --- | --- | --- | --- |
| accuracy | Distractor | 56.88 (16.16) | 46.89 (11.40) | 1.43 | .175 | .715 |
|  | Non-distractor | 54.49 (19.06) | 49.0 (12.3) | 0.705 | .492 | .353 |
| percentage of false positives | Distractor | 43.21 (20.31) | 37.99 (18.99) | 0.533 | .603 | .266 |
|  | Non-distractor | 41.5 (19.54) | 41.0 (27.72) | 1.536 | .967 | .021 |
| reaction time in ms | Distractor | 571.51 (81.86) | 585.23 (110.23) | 0.283 | .782 | .141 |
|  | Non-distractor | 604.83 (110.71) | 586.08 (131.34) | 0.309 | .762 | .154 |
| standard deviation of reaction time in ms | Distractor | 151.75 (16.85) | 162.47 (24.64) | 1.016 | .327 | .508 |
|  | Non- distractor | 162.42 (35.71) | 166.39 (27.44) | 0.249 | .807 | .125 |
