# Supplementary material for: The effect of 5-hydroxytryptophan, a serotonin precursor, on adults with high levels of Attention Deficit Hyperactivity Disorder traits: A randomised, controlled trial
Source: PLoS One. 2026 May 20;21(5):e0349512. doi: 10.1371/journal.pone.0349512 (PMC13189352; doi:10.1371/journal.pone.0349512)
Supplement: S2 Table — (DOCX) [file pone.0349512.s007.docx]

# Supporting information:

**Table S7: Flanker performance measures for the participants with ADHD diagnosis and lowest ASRS scorers matched group.**

| Measure | Condition | ADHD group | Low ASRS group | t | p | Cohen’s d |
| --- | --- | --- | --- | --- | --- | --- |
| accuracy | Incongruent | 86.03 (10.04) | 91.07 (6.75) | 1.178 | .259 | .589 |
|  | Congruent | 98.14 (1.73) | 98.23 (1.66) | 0.107 | .916 | .054 |
| reaction time (ms) | Incongruent | 453.45 (50.90) | 506.42 (59.99) | 1.904 | .078 | .952 |
|  | Congruent | 394.10 (41.43) | 456.76 (38.54) | 3.132 | .007 | 1.566 |
| standard deviation of reaction time (ms) | Incongruent | 97.18 (58.81) | 140.35 (87.52) | 1.158 | .266 | .579 |
|  | Congruent | 83.76 (59.02) | 116.68 (49.78) | 1.598 | .132 | .799 |
