# Supplementary material for: The effect of 5-hydroxytryptophan, a serotonin precursor, on adults with high levels of Attention Deficit Hyperactivity Disorder traits: A randomised, controlled trial
Source: PLoS One. 2026 May 20;21(5):e0349512. doi: 10.1371/journal.pone.0349512 (PMC13189352; doi:10.1371/journal.pone.0349512)
Supplement: S3 Table — (DOCX) [file pone.0349512.s008.docx]

# Supporting information:

**Table S8: mean performance across measures in the flanker and N-back task, split by ADHD subtype.**

| Measure | Condition | Combined-type M(SD) | Inattentive-type M(SD) | Low ASRS group M(SD) | F | P | ηp2 | |
| --- | --- | --- | --- | --- | --- | --- | --- | --- |
| ***N-Back task*** | | | | | | | |  |
| Accuracy | Distractor | 53.57 (16.72) | 45.05 (11.96) | 51.98 (13.11) | 2.355 | .100 | .042 | |
|  | Non-distractor | 54.46 (17.62) | 45.68 (12.74) | 55.73 (17.19) | 2.646 | .076 | .047 | |
| Percentage of false positives | Distractor | 40.96 (16.03) | 37.83 (16.15) | 38.96 (14.68) | 0.302 | .740 | .006 | |
|  | Non-distractor | 45.43 (15.23) | 42.37 (17.00) | 41.20 (17.51) | 0.695 | .501 | .013 | |
| Reaction time | Distractor | 588.48(64.36) | 598.61 (66.21) | 587.73 (75.85) | 0.177 | .838 | .003 | |
|  | Non-distractor | 422.56 (59.75) | 431.59 (48.46) | 427.44 (52.49) | 0.184 | .832 | .003 | |
| Standard deviation of reaction time | Distractor | 172.08 (17.95) | 172.06 (16.87) | 165.05 (20.20) | 1.88 | .158 | .034 | |
|  | Non-Distractor | 177.37 (31.27) | 170.69 (30.74) | 155.14 (25.75) | 7.038 | **.001** | .116 | |
| ***Flanker task*** | | | | | | | |  |
| Accuracy | Incongruent | 87.35 (8.59) | 90.31 (7.04) | 89.49 (7.19) | 1.206 | .303 | .022 | |
|  | Congruent | 97.96 (2.09) | 97.59 (1.68) | 98.05 (1.71) | 0.44 | .645 | .008 | |
| Reaction time | Incongruent | 473.46 (72.88) | 494.64 (62.98) | 481.12 (58.94) | 0.667 | .515 | .012 | |
|  | Congruent | 422.56 (59.75) | 431.59 (48.46) | 427.44 (52.49) | 0.184 | .832 | .003 | |
| Standard deviation of reaction time | Incongruent | 106.60 (42.36) | 127.89 (49.16) | 106.26 (52.95) | 1.503 | .227 | .027 | |
|  | Congruent | 92.97 (35.31) | 103.44 (30.96) | 91.33 (40.22) | 0.767 | .467 | .014 | |
