# Supplementary material for: The effect of 5-hydroxytryptophan, a serotonin precursor, on adults with high levels of Attention Deficit Hyperactivity Disorder traits: A randomised, controlled trial
Source: PLoS One. 2026 May 20;21(5):e0349512. doi: 10.1371/journal.pone.0349512 (PMC13189352; doi:10.1371/journal.pone.0349512)
Supplement: S4 Table — (DOCX) [file pone.0349512.s009.docx]

# Supporting information:

**Table S9: ANOVA results for intervention x timepoint on performance measures in the N-back task.**

| Measure | Condition | Timepoint | 5-HTP M(SD) | Placebo M (SD) | F | p | ηp2 |
| --- | --- | --- | --- | --- | --- | --- | --- |
| Accuracy | Audio | Pre | 50.46 (15.86) | 52.63 (12.78) | 0.493 | .484 | .005 |
|  |  | Post | 54.64 (18.11) | 58.39 (14.51) |  |  |  |
|  | Silent | Pre | 54.09 (18.99) | 53.25 (14.57) | 6.605 | **.012** | .058 |
|  |  | Post | 53.69 (22.10) | 60.82 (20.06) |  |  |  |
| Percentage of false positives | Audio | Pre | 41.76 (16.21) | 36.61 (13.88) | 0.007 | .935 | .000 |
|  |  | Post | 37.43 (16.08) | 32.44 (14.56) |  |  |  |
|  | Silent | Pre | 46.13 (17.41) | 39.32 (15.01) | 0.255 | .614 | .002 |
|  |  | Post | 43.18 (21.69) | 34.79 (18.28) |  |  |  |
| Reaction time (ms) | Audio | Pre | 591.02 (69.38) | 588.11 (70.67) | 0.310 | .579 | .003 |
|  |  | Post | 594.60 (82.50) | 586.31 (78.80) |  |  |  |
|  | Silent | Pre | 601.96(104.69) | 589.30 (88.95) | 0.939 | .335 | .009 |
|  |  | Post | 604.44 (98.36) | 579.09 (87.54) |  |  |  |
| Standard deviation of reaction time (ms) | Audio | Pre | 169.30 (19.53) | 167.60 (18.51) | 0.497 | .482 | .005 |
|  |  | Post | 163.68 (21.27) | 158.82 (20.79) |  |  |  |
|  | Silent | Pre | 169.83 (33.57) | 160.27 (24.78) | 0.062 | .804 | .001 |
|  |  | Post | 167.35 (29.96) | 159.44 (29.41) |  |  |  |
