# Supplementary material for: The effect of 5-hydroxytryptophan, a serotonin precursor, on adults with high levels of Attention Deficit Hyperactivity Disorder traits: A randomised, controlled trial
Source: PLoS One. 2026 May 20;21(5):e0349512. doi: 10.1371/journal.pone.0349512 (PMC13189352; doi:10.1371/journal.pone.0349512)
Supplement: S5 Table — (DOCX) [file pone.0349512.s010.docx]

# Supporting information:

**Table S10:** **ANOVA results for intervention x ASRS group x timepoint on performance measures in the N-back task.**

| Measure | Condition | Timepoint | High ASRS group M (SD) | | Low ASRS group M (SD) | | F | p | ηp2 |
| --- | --- | --- | --- | --- | --- | --- | --- | --- | --- |
|  |  |  | 5-HTP | Placebo | 5-HTP | Placebo |  |  |  |
| Accuracy | Audio | Pre | 52.57 (17.03) | 49.64 (14.20) | 48.36 (14.59) | 55.61 (10.48) | 0.661 | .418 | .006 |
|  |  | Post | 54.96 (19.46) | 55.46 (15.76) | 54.32 (17.01) | 61.32 (12.76) |  |  |  |
|  | Silent | Pre | 53.61 (17.16) | 49.61 (15.66) | 54.60 (20.97) | 56.89 (12.63) | 0.136 | .713 | .001 |
|  |  | Post | 54.78 (21.41) | 57.61 (23.54) | 52.61 (23.10) | 64.04 (15.63) |  |  |  |
| Percentage of false positives | Audio | Pre | 45.86 (14.85) | 32.96 (14.47) | 37.66 (16.73) | 32.96 (14.47) | 1.096 | .297 | .010 |
|  |  | Post | 41.66  (13.64) | 26.93 (13.85) | 33.19 (17.43) | 26.93 (13.85) |  |  |  |
|  | Silent | Pre | 51.00 (13.70) | 37.50 (14.42) | 41.25 (19.51) | 41.14 (15.63) | 0.003 | .959 | .000 |
|  |  | Post | 45.86  (17.21) | 30.93 (20.46) | 40.50 (25.45) | 38.64 (15.21) |  |  |  |
| Reaction time (ms) | Audio | Pre | 579.62 (55.74) | 603.17 (69.72) | 602.42 (80.18) | 603.17 (69.72) | 2.200 | .141 | .020 |
|  |  | Post | 589.05 (73.97) | 592.91 (85.04) | 600.16 (91.27) | 592.91 (85.04) |  |  |  |
|  | Silent | Pre | 607.86 (78.24) | 604.22 (89.63) | 596.07 (127.01) | 574.38 (87.29) | 0.181 | .672 | .002 |
|  |  | Post | 601.45 (87.62) | 590.69 (92.26) | 607.43 (109.6) | 567.50 (82.58) |  |  |  |
| Standard deviation of reaction time (ms) | Audio | Pre | 176.41 (14.59) | 167.29  (18.50) | 162.20 (21.43) | 167.91 (18.84) | 0.409 | .524 | .004 |
|  |  | Post | 166.63 (21.68) | 157.22 (19.49) | 160.73 (20.82) | 160.42  (22.26) |  |  |  |
|  | Silent | Pre | 188.12 (28.52) | 161.81 (26.67) | 151.54 (28.09) | 158.74 (23.12) | 1.625 | .418 | .006 |
|  |  | Post | 175.09 (24.80) | 158.83 (33.81) | 159.62 (33.01) | 160.05 (24.86) |  |  |  |
