# Supplementary material for: The effect of 5-hydroxytryptophan, a serotonin precursor, on adults with high levels of Attention Deficit Hyperactivity Disorder traits: A randomised, controlled trial
Source: PLoS One. 2026 May 20;21(5):e0349512. doi: 10.1371/journal.pone.0349512 (PMC13189352; doi:10.1371/journal.pone.0349512)
Supplement: S6 Table — (DOCX) [file pone.0349512.s011.docx]

# Supporting information:

**Table S11: ANCOVA results for intervention x ASRS group x timepoint on performance measures in the N-back task, age as covariate.**

| Measure | Condition | F | p | ηp2 |
| --- | --- | --- | --- | --- |
|  |  |  |  |  |
| Accuracy | Audio | 0.606 | 0.438 | 0.006 |
|  |  |  |  |  |
|  | Silent | 0.114 | 0.737 | 0.001 |
|  |  |  |  |  |
| Percentage of false positives | Audio | 0.965 | 0.328 | 0.009 |
|  |  |  |  |  |
|  | Silent | 0.004 | 0.949 | <0.001 |
|  |  |  |  |  |
| Reaction time (ms) | Audio | 2.431 | 0.122 | 0.022 |
|  |  |  |  |  |
|  | Silent | 0.089 | 0.766 | 0.001 |
|  |  |  |  |  |
| Standard deviation of reaction time in (ms) | Audio | 0.468 | 0.495 | 0.004 |
|  |  |  |  |  |
|  | Silent | 1.149 | 0.286 | 0.011 |
|  |  |  |  |  |
