# Supplementary material for: The effect of 5-hydroxytryptophan, a serotonin precursor, on adults with high levels of Attention Deficit Hyperactivity Disorder traits: A randomised, controlled trial
Source: PLoS One. 2026 May 20;21(5):e0349512. doi: 10.1371/journal.pone.0349512 (PMC13189352; doi:10.1371/journal.pone.0349512)
Supplement: S7 Table — (DOCX) [file pone.0349512.s012.docx]

# Supporting information:

**Table S12: ANCOVA results for intervention x ASRS group x timepoint on performance measures in the N-back task, Baseline ASRS score as covariate.**

| Measure | Condition | F | p | ηp2 |
| --- | --- | --- | --- | --- |
|  |  |  |  |  |
| Accuracy | Audio | 0.303 | 0.583 | 0.003 |
|  |  |  |  |  |
|  | Silent | 0.104 | 0.748 | 0.001 |
|  |  |  |  |  |
| Percentage of false positives | Audio | 0.664 | 0.417 | 0.006 |
|  |  |  |  |  |
|  | Silent | <0.001 | 0.985 | <0.001 |
|  |  |  |  |  |
| Reaction time (ms) | Audio | 2.509 | 0.116 | 0.023 |
|  |  |  |  |  |
|  | Silent | 0.183 | 0.670 | 0.002 |
|  |  |  |  |  |
| Standard deviation of reaction time (ms) | Audio | 0.707 | 0.402 | 0.007 |
|  |  |  |  |  |
|  | Silent | 2.134 | 0.147 | 0.020 |
|  |  |  |  |  |
