# Supplementary material for: The effect of 5-hydroxytryptophan, a serotonin precursor, on adults with high levels of Attention Deficit Hyperactivity Disorder traits: A randomised, controlled trial
Source: PLoS One. 2026 May 20;21(5):e0349512. doi: 10.1371/journal.pone.0349512 (PMC13189352; doi:10.1371/journal.pone.0349512)
Supplement: S8 Table — (DOCX) [file pone.0349512.s013.docx]

# Supporting information:

**Table S13: ASRS scores of high ASRS group split by diagnosis status.**

|  | ADHD diagnosis self-report (n = 8) | High ASRS with no ADHD diagnosis (N = 47) | T | p |
| --- | --- | --- | --- | --- |
| ASRS screener  M (SD) | 5.38 (0.74) | 4.83 (0.79) | 1.82 | .074 |
| ASRS hyperactive score  M (SD) | 6.62 (2.20) | 4.91 (1.93) | 2.27 | **.027** |
| ASRS inattentive score  M (SD) | 7.88 (1.13) | 7.15 (1.33) | 1.45 | .153 |
