# Supplementary material for: The effect of 5-hydroxytryptophan, a serotonin precursor, on adults with high levels of Attention Deficit Hyperactivity Disorder traits: A randomised, controlled trial
Source: PLoS One. 2026 May 20;21(5):e0349512. doi: 10.1371/journal.pone.0349512 (PMC13189352; doi:10.1371/journal.pone.0349512)
Supplement: S2 Supplement — (DOCX) [file pone.0349512.s015.docx]

## Supplementary information 15: Study protocol approved by Ethical Comittee

Acute 5-HTP loading impact on distractibility in populations with high and low levels of ADHD symptoms.

**Introduction:**

Attention Deficit Hyperactivity Disorder (ADHD) is one of the most common neurodevelopmental disorders worldwide, affecting around 5% of children and 2.5% of the adult population (Polanczyk et al., 2007, 2014; Song et al., 2021). The disorder presents with symptoms of inattention, hyperactivity, and impulsivity, but ADHD individuals also suffer from other risks and functional impairments that ultimately result in a lower quality of life (Orm et al., 2023). Current frontline treatment for ADHD in adults involves treatment with psychostimulants, but estimates of 20-50% of adults are considered nonresponders to such medication, and adults who do respond typically only show a 50% or less reduction in ADHD symptoms (Safren et al., 2007). Furthermore, psychostimulants are controversial given the risk of drug diversion or selling; in 2010 a report established that 61.7% of college students on ADHD medication had diverted medication at least once, many in the form of sharing with friends (Garnier et al., 2010). This is reflected in the 2018 USA National Survey on Drug use and Health, which reported that 5.1 million people over the age of 12 had misused amphetamine or methylphenidate products, both of which are used in the treatment of ADHD (Lipari and Park-Lee, 2019). Combined with the inability of psychostimulants to be used by children with ADHD under 5 years of age, there is a need to find alternative therapies for the treatment of ADHD.

One area of pharmacological interest is the serotonergic system, which has been identified as a system with atypical function in ADHD. Of particular interest is how the metabolic pathway of serotonin (5-HT) synthesis; significant changes to ratios of 5-HT metabolites such as 5-hydroxyindoleacetic acid and homovanillic acid have been found in the CSF and urine of ADHD individuals, as well as some correlation being observed between levels of metabolites and performance in continuous performance tasks (Oades, 2002, 2008). Furthermore, there have been a number of mutations of genes in 5-HT synthesis implicated in ADHD (Faraone & Larsson, 2019). Of specific interest is mutations found in tryptophan hydroxylase (TPH2), the enzyme responsible for the conversion of dietarily obtained tryptophan to 5-hydroxytryptophan (5-HTP), the precursor to serotonin. Not only do TPH2 mutations potentially reduce the amount of serotonin produced in ADHD individuals, the conversion of tryptophan to 5-HTP is already the rate limiting step in serotonin synthesis, potentially exacerbating already reduced levels of serotonin production (Siesser et al., 2013, Akhrif et al., 2023).

Considering that ADHD may be in part due to dysfunction in the serotonergic system, it is logical to consider Selective Serotonin Reuptake Inhibitors (SSRIs) as a potential pharmacotherapy. However, despite strong preclinical rationale for the use of SSRIs in the treatment of ADHD, clinical trials have found conflicting evidence. A few studies have found a moderate to good improvement in ADHD symptoms, with a significant decrease in inattentive symptoms and 47% of patients showing improvement (Barrickman et al., 1991; Quintana et al., 2007). Others however found no clinical benefit (Donnelly et al., 1989), or at worst, an exacerbation of ADHD symptoms (Riddle et al.,1990). There are a number of ideas as to why SSRIs have not been successful in ameliorating ADHD symptoms; some studies may have trial periods which are too short to observe effects of SSRI treatment, for example. It has also been postulated that the activity of the 5-HT_1A_ receptor, which regulates the synaptic availability of 5-HT, needs long periods of drug desensitisation to effectively increase the bioavailability of 5-HT in ADHD trials (Riley and Overton, 2019).

It is well accepted that ADHD is heterogeneous in its symptom presentation, with diagnosis depending on the key symptoms of hyperactivity, impulsivity and distractibility (American Psychiatric Association, 2013). As such, a more useful approach to therapies may involve targeting specific symptoms of the disorder rather than trying to tackle dysfunction as a whole. As such, this research will primarily focus on distractibility, a trait implicated in one of the triumvirate of core ADHD symptoms, inattention. Distractibility and inattention are the most likely ADHD traits to persist into adulthood, suggesting it is one of the more significant symptoms to be tackled (Biederman, 2005). It is possible that distractibility in ADHD is intimately linked with serotonergic activity in subcortical structures such as the superior colliculus, and could also be linked to psychostimulant mechanisms resulting in symptom improvement. For example, methylphenidate administration has been found to ameliorate deficits in motor planning and response inhibition of saccades in people with ADHD, both collicular functions (O’Driscoll et al., 2005). Furthermore, in vitro application of d-amphetamine and methylphenidate have been found to depress stimulus intensity in the superior colliculus, effects that were mimicked by application of 5-HT, and when in particularly high concentrations 5-HT produced a near complete response suppression. Application of the 5-HT antagonist metergoline also blocked the effect of d-amphetamine and methylphenidate, suggesting that psychostimulants may reduce distractibility by altering serotonergic function in the superior colliculus (Dommett et al., 2009).

Considering the existing research on serotonin synthesis, ADHD, attention and existing pharmacotherapies, we hypothesise that a reduction in 5-HTP synthesis may be a cause of reduced 5-HT in ADHD. Mechanistically, this may then cause reduced serotonergic inhibition in the superior colliculus, resulting in increased response to stimuli, and causing increased distractibility. Furthermore, we postulate that 5-HTP supplementation may ameliorate symptoms of ADHD as a result. Limited research has been conducted on the impact of 5-HTP on attention and distractibility, with only one study on rhesus macaques having been completed. This research however demonstrated that 5-HTP modulated attention in these animals in a bi-directional manner, dependent on the macaque’s baseline levels of attention; those with low initial attention had an increase in looking behaviour, those with high initial attention experienced a decrease (Weinberg-Wolf et al., 2018). As such, this research intends to explore the impact of acute 5-HTP administration in distractibility across human populations with high and low levels of ADHD symptoms.

**Study design:**

We will assess our hypotheses by conducting a double-blind trial, recording measures of distractibility pre and post oral 5-hydroxytryptophan administration using a task-relevant and task-irrelevant distractor test. Measures of reaction time and accuracy on these tests will be taken to determine a participant's distractibility. Participants will be stratified into 2 groups with high and low levels of ADHD symptoms as measured using the ASRS v1.1 ADHD screener. The study will use an acute, high dose administration of 5-HTP or placebo, which will be administered to the two groups in a 1:1 ratio. Assignment to intervention or placebo groups will be randomised. This will be a 2 (intervention: placebo or 5-HTP) x 2 (group: high ADHD symptoms vs low ADHD symptoms) by 2 (time: pre and post administration) design.

**Hypotheses:**

We hypothesise that:

1. Accuracy in intra-task and external-task distractors will be lower for participants with a higher score on the ASRS v1.1 ADHD screener, whereas reaction time will be higher.
2. 5-HTP administration will improve reaction time and accuracy in internal-task and external-task distractor tests for participants with high scores on the ASRS.

**Participant recruitment:**

Participants will be recruited from the University of Sheffield volunteers database, local community groups around Sheffield and the university’s student population using posters, emails and advertisements on websites, newsletters and social media pages. All recruitment material will include a link to the study’s information sheet and a consent form for interested individuals, whereby they will be able to complete an online pre-screening survey. Upon recruitment, participants will be invited to book a 2.5 hour slot at the university to participate in the trial. Participants who attend the experiment will be compensated for their time with £30 amazon vouchers.

No prior research is currently available measuring the impact of 5-HTP on distractibility, meaning that we were unable to use pre existing data to inform power analysis and sample size. A priori power analysis for repeated measures ANOVA were completed using G-power (Faul et al., 2009), and calculated a participant number of 112 for a medium effect size (two tailed, alpha=0.05, beta = 0.08, effect size= 0.3). Given the time constraints of the project and previous difficulty in recruiting participants with high ADHD symptoms, this value may not be reached. For this sake, we will aim to recruit at least 100 participants to participate in the study. This will give 4 groups of 25 participants in a 2 (High and low ADHD symptom scores) x 2 (5-HTP or placebo) design.

Participant Eligibility:

*Inclusion criteria.*

Participants will be between the ages of 18-65 years of age, with the lower age limit to ensure only the target population of adults is assessed, and the upper age limit based on the working age in the UK. Control participants will be selected based on a score of 1 or less on the Adult ADHD self-report scale screener (ASRS v1.1, items 1-6) (Kessler et al., 2005). Participants in the high ADHD symptoms will be required to have a score of over 4 on the ASRS v1.1, the score found to be highly consistent with ADHD. In an attempt to widen participation criteria to subclinical presentation of ADHD, participants will not be required to have a clinical diagnosis, only a score in the ASRS indicative of symptoms highly consistent with ADHD.

*Exclusion criteria.*

Participants will be excluded if they are taking medication for treatment of ADHD, so as to understand the effect of 5HTP solely, without the interference of psychostimulant action. Participants who use other medications known to impact the serotonergic system such as Selective Serotonin Reuptake Inhibitors will also be excluded. The 5-HTP supplementation used in the study does not contain any of the 14 known allergens, but the placebo contains lactose, meaning vegans and individuals with lactose intolerance or milk allergies will be excluded. Other exclusion criteria will include pregnant and breastfeeding women; at current it is unknown what effect 5-HTP has on pregnant women. Smokers will also be excluded. Participants with psychological or neurological disorders or patients taking any other psychoactive medications will also be excluded from the study. In order to ensure other factors influencing reaction time and accuracy within tasks are minimised, participants with dyslexia will be excluded.

**Methods and Materials:**

Online prescreen:

The online screening survey will include items to assess whether a respondent meets the age, ADHD symptom and medication use inclusion criteria. In the interest of keeping the pre-screen questionnaire as brief as possible, participants will be asked a minimum of demographic information at this time, asking age and sex assigned at birth. The ASRS v1.1 will be used to assess ADHD symptoms. Results from the prescreen will be reviewed by the lead researcher within 5 days of their completion, and respondents will be emailed. Eligible participants will be contacted by email and sent a link to identify a suitable time for them to attend the University of Sheffield for a 2.5 hour testing slot. Participants will be sent a reminder of the session 36 hours prior, and reminded of the requirements to complete a 48 hour food diary and fast for 2 hours prior to the session. A template form for the digital food diary will be provided for participants.

*ASRS v1.1:*

In the prescreen, the 6-question ASRS v1.1 (Kessler et al., 2005) screener will be used, as a well validated clinical tool for pre-screening ADHD. Participants are requested to answer the questions with regard to their behaviour over the preceding 6 months. Participants will answer questions using the following likert rating scale: never, rarely, sometimes, often or very often. On questions 1-3, a score of one point is given for answers of sometimes, often or very often. The remaining questions give a score of 1 point for answers of often or very often. Any other answer is given 0 points. A score of 4 or more is considered as symptoms highly consistent with an ADHD diagnosis, so a group with high levels of ADHD symptoms will be defined as participants with a score of 4 or more. Reports of ASRS scores in the US found a mean score of 2.0, so participants with low levels of symptoms will be defined as participants with a score of 1 or less (Adler et al., 2018). Participants performing the pre-screen with scores outside of these ranges will not be invited to participate in the study.

Although the screener will be used to determine participant eligibility, participants will also be asked to complete the remaining 12 questions which make up the complete extended 18 question ASRS v1.1 whilst completing the prescreen (Kessler et al., 2005). Questions will be split into sections assessing inattention and hyperactivity-impulsivity, which will allow assignment of participants to the subtype of ADHD which they present most similarly to. Participants will answer questions using the following rating scale: never, rarely, sometimes, often or very often. On questions 3 ,4 ,5 ,9 ,11 ,17 and 18 a score of one point is given for answers of sometimes, often or very often. All other questions give a score of 1 point for often or very often. Other answers are assigned zero points.

Pre test arrangements:

Participants will be asked to keep a food diary using a digital link for the 48 hours preceding the study, including note of any medications or psychologically active substances such as alcohol and nicotine they have taken during this time period. On the day of the participants' test, participants will abstain from consuming alcohol, caffeinated drinks or nicotine (i.e., smoking, vaping, using nicotine gum or nicotine patches). Participants will also be asked to fast for 2 hours prior to the study, only consuming water in this period, to control for intake of tryptophan and subsequent metabolism of it.

Randomisation:

Participants will partake in a double-blinded, randomised experiment. Upon registering for the study participants will be assigned a participant number that their data will be linked to for the entirety of the study. Using a random number generator, randomisation will be stratified by group (high-ADHD and low-ADHD symptoms) ADHD subtype for those within the high-ADHD symptom group (inattentive, hyperactive and combined type), with a 1:1 allocation to intervention and placebo conditions, recorded as A or B.

In order to blind the researcher delivering the intervention, envelopes containing the doses of placebo and 5-HTP will be labelled as A and B by an assisting researcher, and the contents of A and B will be written down and sealed in an envelope to be opened once the study is completed. Participants will be blind as to whether they are receiving 5-HTP or placebo. Task order will also be randomised for each participant, using a randomising web application. The random number list used to create four blocks of 25 participants (High ADHD and 5-HTP, high ADHD and placebo, Low ADHD and 5-HTP, low ADHD and placebo) will also be created using the web applications available at http://random.org.

Dosage of 5-HTP:

Participants in the intervention group will be given a 200mg dose of 5-HTP administered orally, based on dosage in 5-HTP studies for depression and a recent study looking at acute 5-HTP administration and social cognition (Zamoscik et al., 2021). 5-HTP will be provided by Nature’s best supplement company, in a tablet that contains 3982mg of griffonia seed extract, providing 100mg of 5-HTP. The tablets consist of calcium carbonate, griffonia seed extract, Anti-caking Agents (Silicon Dioxide, Stearic Acid & Magnesium Stearate) and Tablet Coating (Hydroxypropyl Methylcellulose, Glycerol). The supplement provided is vegan and free from wheat, yeast, dairy, soya, nuts, shellfish and gluten containing ingredients. Participants will be asked to inform the researchers if they have any allergies which may impact their reaction to 5-HTP or placebo prior to their participation. The placebo tablets will be provided by Ainsworth’s homoeopathic remedies, and will be unmedicated lactose/sucrose tablets. Due to containing lactose, these tablets are not suitable for participants with a vegan diet or those with lactose intolerance. This will be highlighted in the study prescreen.

Participants will be observed taking 2 of these tablets, or 2 placebo tablets, with a glass of water. Participants will be then asked to wait for 90 minutes in a controlled environment prior to repeating the distractor tests they completed prior to intervention. Participants will be allowed to spend this time at leisure, but must remain under observation to ensure that no food is eaten within this period. Participants will be allowed water.

Flanker test:

Participants will be asked to complete an adapted version of the Eriksen flanker test (Eriksen and Eriksen, 1974). A flanker has been chosen as a test of behavioural inhibition to irrelevant information, and is considered a reliable test of response to in-test distractors. There is significant evidence that ADHD individuals experience increased reaction time and reduced accuracy on such tests compared to a typically developed population, meaning performance should differ between ADHD high and low groups (Mullane et al., 2009). Furthermore, there is evidence to suggest that remission in ADHD symptoms due to ageing is linked to improved performance in the flanker test (Michelini et al., 2016.)

Both the flanker test and task-irrelevant distractor will be built using Psychopy, a python based programme for building psychological experiments (Pierce et al.,2019). In the flanker test, participants will be asked to fixate on a fixation cross in the middle of a monitor. They will be presented with a series of 5 chevrons, and will be asked to report the direction of the middle chevron using a key press (Z for left, M for right). Chevrons will either be presented in a congruent manner facing left (<<<<<), congruent facing right (>>>>>), incongruent left (>><>>) or incongruent right (<<><<). The chevrons will display on the screen for 200ms, and a further 1000 ms allowed to elapse on the fixation screen prior to another trial. Participants will be given a 20 trial practice block to complete with feedback prior to completing the test phase. 100 of each condition (400 total) will be arranged randomly across 10 blocks, allowing participants to take a break, with the testing phase taking approximately 16 minutes, accounting for a 30 second break between each testing block. Participants reaction time per trial, error rate in congruent trials and error rate in incongruent trials will be recorded, and mean reaction time and accuracy as a percentage calculated.

Task-irrelevant distractor test:

Participants will further complete a task-irrelevant distractor test, aimed to look at participants' distractibility to stimuli extraneous to the task. Again, such studies have found inflated scores in reaction time in ADHD individuals or participants with increased ADHD symptoms (Forster and Lavie, 2016, van der Stigchel et al., 2007), although there is some debate as to whether this is due to an increased response to irrelevant stimuli or other parts of the attentional response. Similar changes in response occur in oddball paradigm tasks, where novel auditory stimuli are presented (Wetzel, Scharf and Widmann 2019, Escera et al., 2000). In order to ensure the task was sufficiently difficult, we decided to use a version of the n-back test,, with interruption from auditory stimuli at several points. There is strong evidence that individuals with ADHD perform significantly poorer on the n-back test compared to controls, and that existing ADHD drugs methylphenidate and atomoxetine help to ameliorate this deficit (Kowalczyk et al.,2023).

To assess this, participants will be asked to focus on a fixation cross for 600ms, and then presented with a letter (H, K, N, W and Z based on the letters from the Eriksen Flanker (Eriksen and Eriksen, 1974). A further 600ms will be allowed to elapse before the fixation cross re-appears, giving a 1000ms window for participants to react. Participants will be asked to recall if the letter they are presented with matches the letter they observed 2 trials ago, and to answer in the affirmative by pressing the spacebar. The study consists of 5 blocks, each presenting 100 letters, 20 of which are targets. On 25% of trials, randomly interspersed throughout the last 5 testing blocks, a 200ms, 85dB noise presents simultaneously with a trial. Randomisation is stratified so that the noise appears on 25% of cues, 25% of targets and 25% of non-target and non-cue letters. Participants will be given a 25 trial practice block with feedback, followed by one block with no auditory stimulus, in order to get a baseline accuracy and reaction time for each participant. Participants will be informed that their reaction time and accuracy are being recorded, and to ignore any noise that they may hear during the duration of the experiment. Blocks laste 160 seconds each, so with 5 blocks, a 40 second practice block and assumed 30 seconds break between each block, the task will take around 16 minutes to complete per participant.

**Experimental procedure**:

Upon arrival, participants will be asked to complete a further consent form and informed that they can withdraw consent at any point during the study. Participants will then be seated in a quiet room with a 22” monitor and keyboard, with seating positioned so their head is 57cm from the monitor and the centre of the screen is at eye level to allow a 1 degree visual angle per cm on the screen. Task order will be randomised per participant using a randomiser application, as mentioned previously. Both tasks will take approximately 15 minutes each to complete, and participants will be offered a short break between tasks. Baseline tasks are expected to take around 35 minutes to complete.

Following completion of the initial battery, participants will be provided with either 200mg 5-HTP (See dosage section) or placebo with a glass of water and observed as they take the tablets. Participants will then be asked to wait for 90 minutes in a waiting room, as 90-120 minutes will lead to the maximal level of 5-HTP in serum and cerebro-spinal fluid based on pharmacokinetic reports (Magnussen and Van Woert,1982). Participants will be allowed to participate in a leisure activity of their choosing whilst they wait.

After the 90 minute waiting period has elapsed, participants will return to the quiet room and complete the two distractibility tests again, in the exact same manner as in the pre-administration test phase. Upon completion, participants will be debriefed, provided with researcher contact details to follow the study, offered a snack given they have fasted for a significant period, and allowed to leave. The experimental procedure will take approximately two hours and 40 minutes to complete per participant.

**Statistical analysis:**

All analyses will be performed with a two-tailed significance value of 5%, and data will be checked for normality using histograms prior to statistical analysis. Reaction time and accuracy in each task will be analysed via a 2 intervention type (5-HTP and placebo) x 2 participant group (high and low ADHD group) x 2 time period (before and after supplement administration) in an ANOVA. We will thus be able to assess differences between groups and if the 5-HTP administration meets our hypotheses.

As we will collect data on medication status and ADHD presentation type in the group with high ADHD scores, we hope to further be able to assess within group differences for subtype of ADHD. A student's t-test may be used to look at differences between medication status, and ANOVA for differences between ADHD subtypes.

**Study limitations:**

Although the ASRS is a well established diagnostic tool, a better standard of reporting would be the use of clinically validated assessments. Due to both time and financial constraints, this study opted for the ASRS v1.1. We also wished to stratify by sex, but due to recruiting and testing occuring over a prolonged period, this will not be possible to do in the initial grouping, and instead any sex differences will need to be assessed in the statistical analysis period. Furthermore, the use of a task irrelevant distractor has been debated as to its influence on ADHD individuals, so may provide unexpected results. We however still chose to use this to provide a complete picture on distractor interference and how this is modulated by 5-HTP intervention.

**References:**

Adler, LA, Faraone, SV, Sarocco, P, Atkins, N, Khachatryan, A. Establishing US norms for the Adult ADHD Self-Report Scale (ASRS-v1.1) and characterising symptom burden among adults with self-reported ADHD. Int J Clin Pract. 2019; 73:e13260. <https://doi.org/10.1111/ijcp.13260>

Akhrif, A., Romanos, M., Peters, K., Furtmann, A. K., Caspers, J., Lesch, K. P., Meisenzahl-Lechner, E. M., & Neufang, S. (2023). Serotonergic modulation of normal and abnormal brain dynamics: The genetic influence of the TPH2 G-703T genotype and DNA methylation on wavelet variance in children and adolescents with and without ADHD. *PLoS ONE*, *18*(4 April). <https://doi.org/10.1371/journal.pone.0282813>

American Psychiatric Association. (2013). *Diagnostic and Statistical Manual of Mental Disorders* (5th Edition). American Psychiatric Association.

Barrickman, L., Noyes, R., Kuperman, S., Schumacher, E., & Verda, M. (1991). Treatment of ADHD with fluoxetine: A preliminary trial. *Journal of the American Academy of Child & Adolescent Psychiatry*, *30*(5), 762–767. <https://doi.org/10.1016/S0890-8567(10)80011-5>

Biederman, J. (2005). Attention-deficit/hyperactivity disorder: A selective overview. In *Biological Psychiatry* (Vol. 57, Issue 11, pp. 1215–1220). <https://doi.org/10.1016/j.biopsych.2004.10.020>

Dommett, E. J., Overton, P. G., & Greenfield, S. A. (2009). Drug therapies for attentional disorders alter the signal-to-noise ratio in the superior colliculus. *Neuroscience*, *164*(3), 1369–1376. <https://doi.org/10.1016/j.neuroscience.2009.09.007>

Donnelly, M., Rapoport, J. L., Potter, W. Z., Oliver, J., Keysor, C. S., Murphy, D. L., Psychiatry Branch Donnelly, C., & of Clinical Science Potter, L. (1989). *Fenfluramine and Dextroamphetamine Treatment of Childhood Hyperactivity Clinical and Biochemical Findings*. <https://jamanetwork.com/>

Eriksen, B., & Eriksen, C. W. (1974). Effects of noise letters upon the identification of a target letter in a nonsearch task*. In *Perception & Psychophysics* (Vol. 16, Issue 1).

Escera, C., Alho, K., & Schröger D Istvá N Winkler, E. (2000). Involuntary Attention and Distractibility as Evaluated with Event-Related Brain Potentials. In *Audiol Neurootol* (Vol. 5). [www.karger.com](http://www.karger.com)

Faraone, S. V., & Larsson, H. (2019). Genetics of attention deficit hyperactivity disorder. In *Molecular Psychiatry* (Vol. 24, Issue 4, pp. 562–575). Nature Publishing Group. <https://doi.org/10.1038/s41380-018-0070-0>

Faul, F., Erdfelder, E., Buchner, A., & Lang, A.-G. (2009). Statistical power analyses using G*Power 3.1: Tests for correlation and regression analyses. *Behavior Research Methods*, *41*, 1149-1160

Garnier, L. M., Arria, A. M., Caldeira, K. M., Vincent, K. B., O’Grady, K. E., & Wish, E. D. (2010). Sharing and selling of prescription medications in a college student sample. *Journal of Clinical Psychiatry*, *71*(3), 262–269. <https://doi.org/10.4088/JCP.09m05189ecr>

Kessler, R. C., Adler, L., Ames, M., Demler, O., Faraone, S., Hiripi, E., Howes, M. J., Jin, R., Secnik, K., Spencer, T., Ustun, T. B., & Walters, E. E. (2005). The World Health Organization adult ADHD self-report scale (ASRS): A short screening scale for use in the general population. *Psychological Medicine*, *35*(2), 245–256. <https://doi.org/10.1017/S0033291704002892>

Kowalczyk, O. S., Cubillo, A. I., Criaud, M., Giampietro, V., O’Daly, O. G., Mehta, M. A., & Rubia, K. (2023). Single-dose effects of methylphenidate and atomoxetine on functional connectivity during an n-back task in boys with ADHD. Psychopharmacology, 240(10), 2045–2060. <https://doi.org/10.1007/s00213-023-06422-7>

Lipari, R. N., & Park-Lee, E. (2019). *Key Substance Use and Mental Health Indicators in the United States: Results from the 2018 National Survey on Drug Use and Health*. <https://www.samhsa.gov/data/>

Magnussen, I., & van Woert, M. H. (1982). Human Pharmacokinetics of Long Term 5-Hydroxytryptophan Combined with Decarboxylase Inhibitors. *Eur J Clin Pharmacol*, *23*, 81–86.

Michelini, G., Kitsune, G. L., Cheung, C. H. M., Brandeis, D., Banaschewski, T., Asherson, P., McLoughlin, G., & Kuntsi, J. (2016). Attention-Deficit/Hyperactivity Disorder Remission Is Linked to Better Neurophysiological Error Detection and Attention-Vigilance Processes. *Biological Psychiatry*, *80*(12), 923–932. https://doi.org/10.1016/J.BIOPSYCH.2016.06.021

Mullane, J. C., Corkum, P. v., Klein, R. M., & McLaughlin, E. (2009). Interference control in children with and without ADHD: A systematic review of flanker and simon task performance. In *Child Neuropsychology* (Vol. 15, Issue 4, pp. 321–342). <https://doi.org/10.1080/09297040802348028>

Oades, R. D. (2002). Dopamine may be “hyper” with respect to noradrenaline metabolism, but “hypo” with respect to serotonin metabolism in children with attention-deficit hyperactivity disorder. *Behavioural Brain Research*, *130*, 97–102.

Oades, R. D. (2008). Dopamine-serotonin interactions in attention-deficit hyperactivity disorder (ADHD). In *Progress in Brain Research* (Vol. 172, pp. 543–565). <https://doi.org/10.1016/S0079-6123(08)00926-6>

O’Driscoll, G. A., Dépatie, L., Holahan, A. L. v., Savion-Lemieux, T., Barr, R. G., Jolicoeur, C., & Douglas, V. I. (2005). Executive Functions and Methylphenidate Response in Subtypes of Attention-Deficit/Hyperactivity Disorder. *Biological Psychiatry*, *57*(11), 1452–1460. <https://doi.org/10.1016/J.BIOPSYCH.2005.02.029>

Orm, S., Øie, M. G., Fossum, I. N., Fjermestad, K., Andersen, P. N., & Skogli, E. W. (2023). Predictors of Quality of Life and Functional Impairments in Emerging Adults With and Without ADHD: A 10-Year Longitudinal Study. *Journal of Attention Disorders*, *27*(5), 458–469. <https://doi.org/10.1177/10870547231153962>

Peirce, J. W., Gray, J. R., Simpson, S., MacAskill, M. R., Höchenberger, R., Sogo, H., Kastman, E., Lindeløv, J. (2019). PsychoPy2: experiments in behavior made easy. Behavior Research Methods. 10.3758/s13428-018-01193-y

Polanczyk, G., Silva de Lima, M., Lessa Horta, B., Biederman, J., & Augusto Rohde, L. (2007). Article The Worldwide Prevalence of ADHD: A Systematic Review and Metaregression Analysis. In *Am J Psychiatry* (Vol. 164).

Polanczyk, G. V., Willcutt, E. G., Salum, G. A., Kieling, C., & Rohde, L. A. (2014). ADHD prevalence estimates across three decades: An updated systematic review and meta-regression analysis. *International Journal of Epidemiology*, *43*(2), 434–442. <https://doi.org/10.1093/ije/dyt261>

Quintana, H., Butterbaugh, G. J., Purnell, W., & Layman, A. K. (2007). Fluoxetine monotherapy in attention-deficit/hyperactivity disorder and comorbid non-bipolar mood disorders in children and adolescents. *Child Psychiatry and Human Development*, *37*(3), 241–253. <https://doi.org/10.1007/s10578-006-0032-7>

Riddle, M. A., King, R. A., Hardin, M. T., Scahill, L., Ort, S. I., Chappell, P., Rasmusson, A. N. N., & Leckman, J. F. (1990). Behavioral Side Effects of Fluoxetine in Children and Adolescents. *Journal of Child and Adolescent Psychopharmacology*, *1*(3), 193–198. <https://doi.org/10.1089/cap.1990.1.193>

Riley, T. B., & Overton, P. G. (2019). Enhancing the efficacy of 5-HT uptake inhibitors in the treatment of attention deficit hyperactivity disorder. *Medical Hypotheses*, *133*. <https://doi.org/10.1016/j.mehy.2019.109407>

Safren, S. A., Duran, P., Yovel, I., Perlman, C. A., & Sprich, S. (2007). Medication adherence in psychopharmacologically treated adults with ADHD. *Journal of Attention Disorders*, *10*(3), 257–260. <https://doi.org/10.1177/1087054706292165>

Siesser, W. B., Sachs, B. D., Ramsey, A. J., Sotnikova, T. D., Beaulieu, J. M., Zhang, X., Caron, M. G., & Gainetdinov, R. R. (2013). Chronic SSRI treatment exacerbates serotonin deficiency in humanized Tph2 mutant mice. *ACS Chemical Neuroscience*, *4*(1), 84–88. <https://doi.org/10.1021/cn300127h>

Song, P., Zha, M., Yang, Q., Zhang, Y., Li, X., & Rudan, I. (2021). The prevalence of adult attention-deficit hyperactivity disorder: A global systematic review and meta-analysis. *Journal of Global Health*, *11*, 1–9. <https://doi.org/10.7189/jogh.11.04009>

Van der Stigchel, S., Rommelse, N. N. J., Deijen, J. B., Geldof, C. J. A., Witlox, J., Oosterlaan, J., Sergeant, J. A., & Theeuwes, J. (2007). Oculomotor capture in ADHD. *Cognitive Neuropsychology*, *24*(5), 535–549. <https://doi.org/10.1080/02643290701523546>

Weinberg-Wolf, H., Fagan, N. A., Anderson, G. M., Tringides, M., Dal Monte, O., & Chang, S. W. C. (2018). The effects of 5-hydroxytryptophan on attention and central serotonin neurochemistry in the rhesus macaque. *Neuropsychopharmacology*, *43*(7), 1589–1594. <https://doi.org/10.1038/s41386-017-0003-7>

Wetzel, N., Scharf, F., & Widmann, A. (2019). Can’t Ignore—Distraction by Task-Irrelevant Sounds in Early and Middle Childhood. *Child Development*, *90*(6), e819–e830. <https://doi.org/10.1111/cdev.13109>

Zamoscik, V., Schmidt, S. N. L., Bravo, R., Ugartemendia, L., Plieger, T., Rodríguez, A. B., Reuter, M., & Kirsch, P. (2021). Tryptophan-enriched diet or 5-hydroxytryptophan supplementation given in a randomised controlled trial impacts social cognition on a neural and behavioural level. *Scientific Reports*, *11*(1). [https://doi.org/10.1038/s41598-021-01164-](https://doi.org/10.1038/s41598-021-01164-y)

[y](https://doi.org/10.1038/s41598-021-01164-y)
